# Mechanistic insights into effects of the cardiac myosin activator omecamtiv mecarbil from mechanokinetic modelling

**DOI:** 10.3389/fphys.2025.1576245

**Published:** 2025-04-17

**Authors:** Alf Månsson

**Affiliations:** Department of Chemistry and Biomedical Sciences, Linnaeus University, Kalmar, Sweden

**Keywords:** omecamtiv mecarbil, heart, cardiac muscle, β-myosin, mechanokinetic model, striated muscle

## Abstract

**Introduction:**

Small molecular compounds that affect the force, and motion-generating actin-myosin interaction in the heart have emerged as alternatives to treat or alleviate symptoms in severe debilitating conditions, such as cardiomyopathies and heart failure. Omecamtiv mecarbil (OM) is such a compound developed to enhance cardiac contraction. In addition to potential therapeutic use, its effects may help to elucidate myosin energy transduction mechanisms in health and disease and add insights into how the molecular properties govern contraction of large myosin ensembles in cardiac cells. Despite intense studies, the effects of OM are still incompletely understood.

**Methods:**

Here we take an *in silico* approach to elucidate the issue. First, we modify a model, previously used in studies of skeletal muscle, with molecular parameter values for human ventricular β-myosin to make it useful for studies of both myosin mutations and drugs. Repeated tests lead to at a set of parameter values that allow faithful reproduction of range of functional variables of cardiac myocytes. We then apply the model to studies of OM.

**Results and discussion:**

The results suggest that major effects of OM such as large reduction of the maximum velocity with more limited effects on maximum isometric force and slowed actin-activated ATPase can be accounted for by two key molecular effects. These encompass a reduced difference in binding free energy between the pre- and post-power-stroke states and greatly increased activation energy for the lever arm swing during the power-stroke. Better quantitative agreement, e.g., isometric force minimally changed from the control value by OM is achieved by additional changes in model parameter values previously suggested by studies of isolated proteins.

## 1 Introduction

Muscle as well as non-muscle cells produce force by cyclic interactions between myosin II motor domains and actin filaments powered by the turn-over of MgATP. It has been demonstrated in recent decades that many different point mutations in myosin II as well as other sarcomere proteins cause deleterious diseases in both skeletal and cardiac muscle. Most common of these are hypertrophic (HCM) and dilated (DCM) cardiomyopathy, where HCM is the leading cause of sudden cardiac death in young people (Maron, 2018; Spudich, 2019; Yotti and Seidman, 2019). Another serious cardiac condition with poor prognosis, is heart failure being a frequent reason for hospitalization. It has a multitude of underlying causes such as myocardial infarction and hypertension but also cardiomyopathies. A common denominator is that the heart fails as a pump, i.e., its pumping work is not sufficient to meet the demands of different organs.

Recently, efforts have been devoted to developing myosin-active drugs that modify contraction in the mentioned diseases to prevent or reverse the pathologic remodeling of the heart (Malik et al., 2011; Green et al., 2016; Spertus et al., 2021). In addition, efforts have been made to develop myosin-active drugs in diseases involving skeletal muscle, e.g., spasticity (Gyimesi et al., 2020). Key beneficial effects of the myosin-active compounds may be attributed to secondary effects such as modulated thick and thin filament-based activation (Nagy et al., 2015; Anderson et al., 2018; Kampourakis et al., 2018; Rohde et al., 2018; Gollapudi et al., 2021). However, recent studies indicate that the direct effects of the drugs on each motor domain are equally or more important (Chu et al., 2021; Mohran et al., 2024). In accordance with the central importance of the release of inorganic phosphate (Pi) from the myosin active site in energy transduction (Robert-Paganin et al., 2020; Debold, 2021; Månsson et al., 2023; Rassier and Månsson, 2025) it is not unexpected that most myosin-active small-molecular compounds developed thus far directly or indirectly affect this transition [e.g., (Kovacs et al., 2004; Liu et al., 2015; Kawas et al., 2017; Rohde et al., 2017; Swenson et al., 2017; Rohde et al., 2018)].

One myosin-active compound recently approved for use in HCM is mavacamten (MAVA) (Green et al., 2016; Spertus et al., 2021). Another compound, omecamtiv mecarbil (OM), was developed for intended use in systolic heart failure (Cleland et al., 2011; Malik et al., 2011). These compounds are interesting because both bind in the same pocket of the myosin motor domain (Auguin et al., 2024) while having different effects on cardiac muscle force. MAVA thus reduces force (Green et al., 2016; Scellini et al., 2021) whereas OM generally has the opposite effect (Malik et al., 2011; Scellini et al., 2024). On the other hand, both compounds reduce the maximum unloaded shortening velocity (or *in vitro* gliding velocity) (Kawas et al., 2017; Swenson et al., 2017) and the actin-activated myosin ATPase in solution (Kawas et al., 2017; Swenson et al., 2017). The effects of these drugs have been associated with different kinetic mechanisms on the molecular level. Whereas there is evidence that MAVA reduces the rate of Pi-release in solution kinetics studies (Kawas et al., 2017; Rohde et al., 2018) OM appears to have the opposite effect (Liu et al., 2015; Rohde et al., 2017). Finally, there is convincing evidence that OM greatly inhibits the swing of the myosin lever arm (Rohde et al., 2017; Woody et al., 2018) usually (as here) denoted “the power stroke.” Thus, somewhat counterintuitively it seems that a compound developed as a myosin activator, inhibits the main force-, and motion-generating step in the motor protein.

The ensemble contractile and kinetic effects (on force, velocity and ATPase) due to MAVA and OM are quite well-characterized. There is less consensus about how the molecular mechanisms suggested by studies of isolated proteins (e.g., for OM) (Malik et al., 2011; Liu et al., 2015; Winkelmann et al., 2015; Planelles-Herrero et al., 2017; Rohde et al., 2017; Swenson et al., 2017; Liu et al., 2018; Woody et al., 2018; Auguin et al., 2024) account for the ensemble effects. Insights in this regard are important in drug discovery because the ensemble effects are central for the clinical use of a drug whereas molecular mechanisms are more readily assessed in early studies using isolated proteins. Bottom-up mechanokinetic modelling where the emphasis is to predict contractile effects on different scales based on kinetic and mechanical characteristics of actomyosin interactions in isolated proteins (“bottom-up”) are helpful in this regard (Eisenberg and Hill, 1978; Månsson et al., 2018; Rahman et al., 2018; Månsson and Rassier, 2022).

Here we first develop and validate a detailed mechanokinetic model that should be generally useful for elucidating drug effects on the actin-myosin interaction. We then focus on elucidating the contractile effects of OM from single molecules to large ensembles. The model was developed from a related model previously used to simulate skeletal muscle contraction including effects of small molecular substances (Rahman et al., 2018; Månsson and Rassier, 2022). It is found to account well for steady-state force-velocity and other ensemble data from experiments on human cardiac cells as well as isolated myosin. The model was then used to evaluate different hypotheses for the origin of the ensemble contractile effects of OM based on molecular mechanisms suggested from a range of previous experimental studies. The results suggest that the major contractile effects of OM can be explained by a strong inhibiting effect of the drug on the power stroke. For better quantitative agreement between model predictions and experimental data it is necessary to also incorporate other previously observed molecular effects of the drug in the simulations. This includes increased affinity in pre-power-stroke states and “overpriming” of the myosin lever arm position in the pre-power-stroke state.

## 2 Materials and methods

### 2.1 Modelling - general

The approaches for modelling follow those in (Rahman et al., 2018; Månsson and Rassier, 2022) for skeletal muscle actomyosin, in turn largely based on ideas developed earlier (Hill, 1974; Eisenberg and Hill, 1978; Eisenberg et al., 1980; Månsson, 2016; 2020).

**FIGURE 1:**
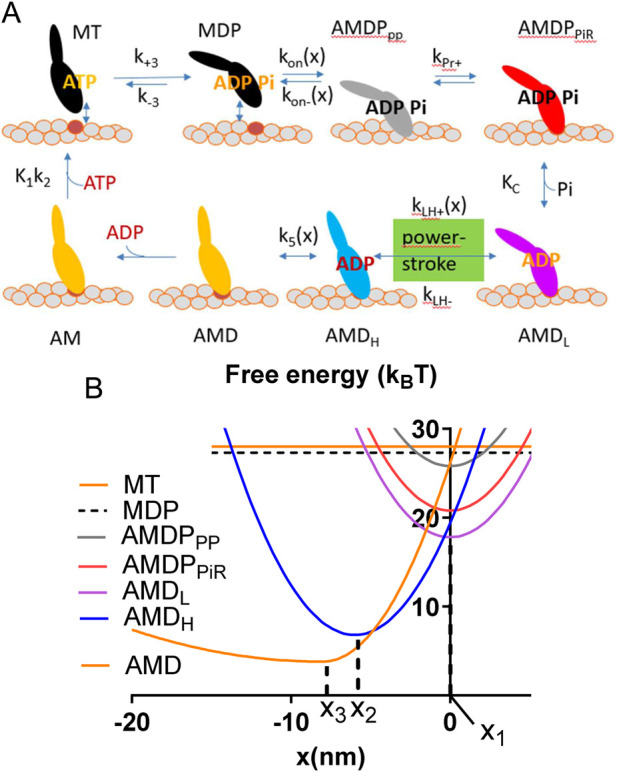
Key transitions and model states. **(A)**. Schematic illustration of myosin head states including their coarse-grained structure in interaction with actin. The model states are encoded by letters where A and M denote actin and myosin respectively and T, D and P denote substrate (ATP) and products (ADP and Pi), respectively (see further text). The subscripts PP and PiR denote a pre-power-stroke state, and a Pi-release state respectively as defined previously (Llinas et al., 2015; Rahman et al., 2018). The subscripts L and H are defined in the text. Upper case and lower-case letters for parameters, refer to equilibrium constants and rate constants, respectively. The argument x indicates strain dependence. **(B)** Free energies of the states defined in A as function of the position coordinate, x where x = 0 nm is defined as the distance between the myosin origin on the thick filament backbone and the actin site where force = 0 pN in the AMDP_PP_ state. Parameters x_1_, x_2_ and x_3_ are indicated.

The model parameters and model structure are defined in Figure 1. Parameter values for the simulations (Tables 1, 2) are based on solution biochemistry and single molecule mechanics studies (Månsson, 2020) subject to limitations discussed below (see also notes of Tables 1, 2). In the modelling we define a position coordinate x as the distance between a myosin head in the pre-power-stroke (AMDP_PP_) state at its minimum free energy (Figure 1) and its nearest actin filament binding site. Key simplifying assumptions include: 1. A uniform distance (x) distribution between the myosin-heads and the closest myosin binding site on actin (Huxley, 1957; Hill, 1974; Månsson, 2010), 2. Only one myosin head available for binding to a given actin site, with independence of the two heads of each myosin molecule and 3. A non-linear (non-Hookean) cross-bridge elasticity using a simplified approach described previously (Månsson et al., 2019). Thus (see also Table 1), stiffness was set to k_s_(x) = 2.8 pN/nm for the AMD and rigor (AM) states at x > x_3_ and to k_s_(x) = 0.2 pN/nm at x < x_3_. For all other states, linear cross-bridge elasticity is assumed with k_s_(x) = 2.8 pN/nm for all x.

**TABLE 1:** Parameter values^a^ for model without OM determining shape of free energy diagrams of (human) cardiac actomyosin.

Parameter	Used values	References data
x_1_ ^b^	0 nm	0 nm
x_2_ ^b^	−6.0 nm^c^	−6.0 nm
x_3_ ^b^	−8.0 nm^c^	−9.0 nm
Δ G_on_ (Free energy difference btw MDP and AMDP_PP_ states)	1.5 k_B_T^d^	k_on_’ exp ( Δ G_on_) ≈11 s^-1^; From (Swenson et al., 2017). 20 mM ionic strength) With k_on_’ = 5 s^-1^; Δ G_on_∼1 k_B_T). The product,. k_on_’ exp ( Δ G_on_) governs actin-activated ATPase (in range 10–15 s^1^; see text) and is important for curvature of force-velocity relationship
ΔΔ G_PiR_ (Free energy difference btw AMDP_PP_ and AMDP_PiR_ states)	1–5 k_B_T^d^ (1 k_B_T eventually chosen)	NA
Δ G_P_ (Free energy difference btw AMDP_PiR_ and AMD_L_ states)	(∼3 k_B_T with current parameters)	See Table 2 (-k_B_T ln ([P_i_]/K_C_))
Δ G_LH_ (AMD_L_- AMD_H_)	11 k_B_T^e^	10 k_B_T
Δ G_HD_ (AMD_H_- AMD)	3 k_B_T^f^	3.5 k_B_T
$\Delta G_{ATP}$	13.1 + ln ([MgATP]/([MgADP][Pi]) k_B_T	28.5 k_B_T (Månsson and Rassier, 2022)
k_s_(x)	2.8 pN/nm (for positive x) 0.2 pN/nm (for negative x for AM and AMD states) 2.8 pN/nm (for negative x for all other states)^g^	2.8 pN/nm (Kaya and Higuchi, 2010) Non-linear elasticity from (Månsson et al., 2019)^g^

^a^ The parameter values are primarily from two-headed myosin motor fragments from porcine cardiac muscle at 26°C, ionic strength <30 mM, pH 7.2. The data from (Hwang et al., 2021) are used instead of human data because they were obtained with full length myosin generally giving higher (and presumably more realistic) (Månsson et al., 2018; Rassier and Månsson, 2025) single molecule mechanics results than data obtained in single motor domains (S1).

^b^ See Figure 1.

^c^ Parameter value modified from experimental data to reasonably account for the force-velocity relationship in the absence of OM as well as the rate of displacement during the myosin power-stroke while also being reasonably consistent with the data in (Hwang et al., 2021) Supplementary Figure S1.

^d^ Values for some parameters could not be found in the literature. They are instead given reasonable values based on previous studies of skeletal muscle (Rahman et al., 2018; Månsson and Rassier, 2022) and to fine-tune the reproduction of experimental data (Supplementary Table S1).

^e^ 11 k_B_T instead of 10 k_B_T for fine tuning of both force-velocity relationship and displacement rate during the power-stroke.

^f^ Somewhat smaller than previously (3 instead of 3.5 k_B_T) to compensate for increase above.

^g^ The simplification with two regions with linear stiffness for positive and negative x-values follow (Månsson et al., 2019). A stiffness value of 0.12 pN/nm was used previously but here a value of 0.2 pN/nm was used for better stability in the numerical computations.

**TABLE 2:** Parameter values^a^ for model in Figure 1 without OM, defining rate functions and kinetic constants for cardiac actomyosin.

Para-meter	Used values	References data and comments
k_+3_ + k_-3_	150 s^−1^ ^b^	14 s^−1^ at 20°C (Bloemink et al., 2014). Assume Q_10_ = 3, 120 mM ionic strength 100 s^−1^ (Bodt et al., 2024) >100 s^−1^. Very short lag in single molecule actin-activated ATPase data (Berg et al., 2024)
K_3_	2	2 in bovine β-myosin; 20 mM ionic strength (Rohde et al., 2017) and porcine β-myosin at < 20 mM IS, 20°C (Liu et al., 2015)
k_on_´	5 s^−1^	11 s^-1^ includes contribution from binding energy, i.e., k_on_ = k_on_’ exp (ΔG_on_) at 20 mM ionic strength and around 25°C. Also limits maximum actin-activated ATPase and rate of Pi release (Stehle, 2017). See also Table 1 for ΔG_on_
k_Pr+_´	3,000 s^-1^	High, not to reduce maximum sliding velocity (Moretto et al., 2022; Caremani et al., 2013)
K_C_	10 mM	∼10 mM in rabbit slow skeletal muscle fibers and guinea pig cardiac myofibrils (Stehle, 2017; Governali et al., 2020)
k_LH-_	5,000 s^-1^	Start with value for power-stroke rate in (Woody et al., 2019; Hwang et al., 2021) (∼1,000 s^−1^) and fine-tune to account for power-stroke rate in simulations (cf. Supplementary Table S1; Supplementary Figure S1)
k_-5_	1,000 s^-1^	Use value for power-stroke rate in (Woody et al., 2019) (∼1,000 s^−1^)
k_6_	100 s^-1^	100–300 s^−1^ at ionic strength <30 mM (Swenson et al., 2017; Tang et al., 2021)
k_adpo_	0–4 s^-1^	ADP dissociation from AMD_L_ state. 10 s^−1^ (Woody et al., 2018)
[Pi]	0.5 mM	0.5 mM (Debold et al., 2011)
[MgATP]	5 mM	Near physiological 5–10 mM (Kuno and Itai, 1992; Kushmerick et al., 1992)
K_1_	1.6 mM^-1^	1.6 mM^−1^ at 20°C. Ionic strength 120 mM (Bloemink et al., 2014)
k_2_	1,400 s^-1^	1,000 s^-^at 20°C and IS 120 mM (Bloemink et al., 2014). Assume Q_10_ = 2. Ionic strength 120 mM

^a^ The parameter values are primarily obtained from myosin motor fragments with heavy chains from human β-cardiac myosin II (but in several cases with murine light chains) at 25°C, ionic strength: ∼30 mM–∼120 mM, pH 7–7.5.

^b^ The value 150 s−1 is used primarily because of the very short initial lag in the dwell-time distribution in single molecule ATPase, assays (Berg et al., 2024).

The rate functions depend on the variable x. First, the transition from the detached MDP state to the first stereo-specifically bound pre-power-stroke (AMDP_PP_) state is governed by:

$$k_{on}\left(x\right)=k_{on}´\;\exp\left(∆G_{\text{on}}–\;\left(k_{s}\left(x\right)/2\right){\left(x‐x_{1}‐\varepsilon \right)}^{2}/\left(2k_{B}T\right)\right)$$ (1)

Here, ΔGon is the difference in free energy minimum between the MDP and AMDP_PP_ states and the parameter ε shifts the maximum of the attachment rate function away from the minimum free energy of the AMDP_PP_ state.

The rate constant for the reversal of this transition depends on x as:

$$k_{\text{on}‐}\left(x\right)=k_{\text{on}}´\;\exp\left({\left(k_{s}\left(x\right)/2)(x‐x_{1‐}\varepsilon \right)}^{2}/\left(2k_{B}T\right)\right)$$ (2)

The attachment step into the AMDP_PP_ state would be expected to occur from a non-specifically weakly bound state AMDP_W_ in rapid equilibrium with the MDP state, rather than directly from the MDP state. It is critically important to take this into account if the aim is to consider the actin-dependence of e.g., steady state ATPase in solution. In the Supplementary Methods we therefore consider the effects of explicitly introducing the weak-binding state but below we neglect it, assuming that the actin concentration is constant (as in the myofilament lattice of muscle) and then assuming that the effects of the rapid pre-equilibrium between the MDP and the AMDP_W_ state is included in the constant k_on_(x) (see further Supplementary Methods).

The subsequent transition into the Pi-release state (AMDP_PiR_) (Llinas et al., 2015; Rahman et al., 2018) and its reversal are governed by:

$${k_{\text{Pr}+}}^{´}\left(x\right)={k_{\text{Pr}+}}^{´}\exp\left(∆G_{\text{PiR}}/2\right)$$ (3)

The reverse rate constant k_LH-_ (Table 2) is independent of x. and

$$k_{\text{Pr}}‐\left(x\right)={k_{\text{Pr}+}}^{´}\exp\left(‐∆G_{\text{PiR}}/2\right)$$ (4)

The quantity, ΔG_PiR_ is the difference between the free energy minima of the AMDP_PP_ and the AMDP_PiR_ states. The next step is the rapid equilibrium for release and re-binding of Pi. We account for this process by lumping together the transition into the Pi-release state and the rapid equilibrium for Pi-release and re-binding. This is achieved by multiplying Equation 4 with the ratio [Pi]/(K_C_ + Pi) where K_C_ is the dissociation constant for Pi-binding to myosin and [Pi] is the solution concentration of inorganic phosphate as follows:

$$k_{\text{Pr}}‐\left(x\right)=\left(\left[\text{Pi}\right]/\left(k_{C}+\text{Pi}\right)\right){k_{\text{Pr}+}}^{´}\;\exp\left(‐∆G_{\text{PiR}}/2\right)$$ (5)

This procedure is approximately valid under the assumption of a constant, low Pi-concentration (<< K_C_) and very high actual Pi-binding and unbinding rate constants [cf. (Dantzig et al., 1992)]. It is not strictly valid for modelling effects of large [Pi] on contractile properties. The rate functions k_Pr+_(x) (Equation 3) and k_Pr-_(x) (Equation 5) are given as functions of x. However, in the present special case where the minimum free energy for both the AMDP and the AMDP_PiR_ states occur at x = x_1_ the rate functions are constants, independent of x.

The next transition in the cycle is the power-stroke [(Huxley and Simmons, 1971) transition; see also (Eisenberg and Hill, 1978)]. The forward transition is given by:

$$\begin{array}{ll} k_{\text{LH}+}\left(x\right) & =k_{\text{LH}-}\exp\left(∆\;G_{\text{LH}\;}+\left(k_{s}\left(x\right)/2\right)\;{\left(x-x_{1}\right)}^{2}/\left(k_{B}T\right)\right.\\ & \;\left.-\left(k_{s}\left(x\right)/2\right){\left(x-x_{2}\right)}^{2}/\left(k_{B}T\right)\right)\;\text{ if }\;k_{\text{LH}+}\left(x\right)\;<\;300000\;s^{-1\;}\text{else }\;k_{\text{LH}+}\left(x\right)\\ & \;=300000\;s^{-1} \end{array}$$ (6)

The transition from the AMD_H_ to the AMD state is governed by:

$$k_{5}\left(x\right)=k_{-5}\exp\left({∆G}_{HD}+\frac{k_{s}\left(x\right){\left(x-x_{2}\right)}^{2}}{2k_{B}T}-\frac{k_{s}\left(x\right){\left(x-x_{3}\right)}^{2}}{2k_{B}T}\right)$$ (7)

where ${∆G}_{HD}$ is the free energy difference between the states. The rate constant for the reverse transition is independent of x and equal to $k_{-5}$

We assume that the ADP-dissociation in the next transition is effectively irreversible (due to very low ADP concentration) occurring with a rate constant k_6_ independent of x (Table 2). The rate constant k_off_ (likewise x-independent) for the subsequent detachment reaction from the AM to the MT state is given by [cf. (Rahman et al., 2018)]:

$$k_{of\;f}=\frac{k_{2}\left[MgATP\right]}{\frac{1}{K_{1}}+\left[MgATP\right]}$$ (8)

Where K_1_ is the equilibrium constant for MgATP binding to the AM state (Figure 1).

### 2.2 Derivation of force-velocity data from state probabilities obtained by solution of differential equations

State probabilities for steady-state muscle contraction at velocity, v, was modeled by solving a set of ordinal differential equations (Hill, 1974) (Equations 9–15):

$$\frac{d\left[MT\right]}{dx}=\left(‐k_{+3}\;\left[\text{MT}\right]+k_{‐3}\left[\text{MDP}\right]+k_{\text{off}}\;\left[\text{AM}\right]+k_{\text{adpo}}\;\left[{\text{AMD}}_{L}\right]\right)/v$$ (9)

$$\frac{d\left[MDP\right]}{dx}=\left(k_{+3}\left[\text{MT}\right]+k_{\text{on}‐}\left(x\right)\left[\text{AMDP}\right]‐\left(k_{\text{on}}\left(x\right)+k_{‐3}\right)\left[\text{MDP}\right]\right)/v$$ (10)

$$\begin{array}{l} \frac{d\left[AMDP\right]}{dx}=(k_{\text{on}}\left(x\right)\left[\text{MDP}\right]+k_{\mathrm{Pr}‐}\left(x\right)\left[{\text{AMD}}_{L}\right]\\ \;‐\left(k_{\text{on}‐}\left(x\right)+k_{\mathrm{Pr}+}\left(x\right))\;\left[\text{AMDP}\right]\right)/v \end{array}$$ (11)

$$\begin{array}{ll} \frac{d\left[{AMD}_{L}\right]}{dx} & =\left(k_{\mathrm{Pr}+}\left(x\right)\;\left[\text{AMDP}\right]+k_{\text{LH}-}\;\left[{AMD}_{H}\right]\right.\\ & \;\left.-\left(k_{\text{LH}+}\left(x\right)+k_{\text{adpo}}+k_{\mathrm{Pr}-}\left(x\right)\right)\;\left[{AMD}_{L}\right]\right)/v \end{array}$$ (12)

$$\begin{array}{ll} \frac{d\left[{AMD}_{H}\right]}{dx} & =\left(k_{\text{LH}+}\;\left(x\right)\left[{AMD}_{L}\right]+k_{‐5}\left[\text{AMD}\right]\right.\\ & \;\left.‒\left(k_{\text{LH}‐}+k_{5}\left(x\right)\right)\right)\left[{AMD}_{H}\right])/v \end{array}$$ (13)

$$\frac{d\left[AMD\right]}{dx}=\left(k_{5}\left(x\right)\left[\;{\text{AMD}}_{L}\;\right]‐\left(k_{6}+k_{‐5}\;\right)\left[\text{AMD}\right]\right)/v$$ (14)

$$\frac{d\left[AM\right]}{dx}=\left(k_{6}\left[\text{AMD}\right]‐{\text{ k}}_{\text{off}}\left[\text{AM}\right]\right)/v$$ (15)

In these equations “ [ ]” indicates probabilities for the different states (Figure 1). Whereas the probabilities vary with x, the argument (x) has been omitted for clarity. Rate functions in the form k_i_(x) are defined in Figure 1, Equations 1–8; Tables 1, 2. The model simulations were performed by numeric solutions (Runge-Kutta-Fehlberg algorithm) of the differential equations using the program Simnon [cf. (Månsson, 2019)]. The observable variables were then calculated from state probabilities (Månsson, 2010) by averaging over the inter-site distance (36 nm) along the actin filament. Thus, average force *<F>* (in pN) per myosin head (whether attached to actin or not) is given by Equation 16:

$$<F>=\sum_{1}^{5}\int_{-91}^{14}k_{s}\left(x\right)\left[A_{j}\right]\left(x\right)\left(x-x_{j}\right)dx/36$$ (16)

Here, the nominator represents summing over all actin-attached cross-bridge states (j = 1 … 5) in Figure 1 with integration over all x-values. The upper integration limit (14 nm) ensures that the entire range for finite probability of cross-bridge attachment to the only available myosin-binding site at the center of a 36 nm actin repeat is covered in the computations. The large negative integration limit x = −91 nm in Equation 16 is a consequence of the assumption of a non-linear cross-bridge elasticity with a very low stiffness of the cross-bridges in the AM and AMD states for negative x. The denominator of 36 nm represents averaging over the 36 nm actin repeat between subsequent binding sites for myosin. To ensure stability in the numerical computations, the values of the rate functions in Equations 1–8 were limited to a maximum (r_max_) of 300,000 s^−1^ and a minimum (r_min_) of 1 10^−6^ s^−1^. If any of the limits was exceeded, the parameter value was set to either r_max_ or r_min_.

With the simulation method used, the maximum isometric force was not the true isometric force but an approximation of this force at a velocity of 1 nm/s (less than 1/1,000 of maximum unloaded shortening velocity) under control conditions In simulations of OM effect the corresponding (approximative zero) velocity was set to 0.1 nm/s due to very low maximum velocity in that case. Lower velocity to estimate isometric force was not possible to use due to instabilities in the numerical computations. However, changes in velocity in the range 0.08–0.5 nm/s suggested negligible underestimation of the isometric force for the OM case.

### 2.3 Simulations of power-stroke

The power-stroke was simulated essentially as in (Moretto et al., 2022) on the assumption that tension is clamped to zero because: 1. It is simplest to assume zero force and 2. It seems likely that the average strain (and thereby force) is close to zero for most cross-bridges attaching into its first pre-power-stroke state in a muscle fiber.

In simulating the power stroke, we used the rate functions above (1–8). Furthermore, consistent with the averaging approach used in single-molecule studies (Woody et al., 2018) and with muscle fiber experiments (Ranatunga et al., 2002), the displacement traces were obtained by solving ordinary differential equations in the state probabilities. Because we are only interested in evaluating the power stroke, we effectively ignored detachment events by setting k_on-_(x) *=* 0 s^−1^ (Equation 2) and [MgATP] = 10 nM. On these assumptions the simulations were performed as follows: Initially, the myosin cross-bridges were assumed to populate an early pre-power-stroke state, i.e., [AMDP_pp_] = 1 (fractional population) whereas the initial values for other state probabilities were set to zero. By assumed clamping of tension to zero, the cross-bridge in the AMDP_PP_ state is held at a strain value x = x_1_ where its free energy is at its minimum. Cross-bridges in the AMDP_PP_ state that progress to the power stroke do this via the AMDP_PiR_ state to the AMD_L_ state (Equation 3). Next, the power stroke occurs with rate constant as in Equation 6, associated with a shift of the cross-bridges along the x-coordinate from x = x_1_ to x = x_2_ under the condition that tension is clamped to zero. The values of the rate functions for transitions from one state into a neighboring state are calculated by inserting a value of *x* in each rate function for which force is zero in the receiving state. Unless otherwise stated we performed the simulations under the assumption of a stiff supporting lattice (as in muscle fibers) or a stiff optical trap (stiffness>> 2.8 pN/nm). Clearly, this is not consistent with most studies such as those of the OM effects in (Woody et al., 2018). We therefore also simulated the conditions with a soft optical trap by changing the cross-bridge stiffness in the model to 0.07 pN/nm.

The displacement time (*t*) course (power stroke progression) of the cross-bridge strain, Δ*L(t)* for a myosin head initially attaching in the AMDP state at *x* = *x*_*1*_ (i.e., with force clamped to zero) is given by Equation 17:

$$\begin{array}{ll} \Delta L\left(t\right) & =\left(\left[{AMD}_{H}\right]\left(t\right)\times \left(x_{1}‐x_{2}\right)+\left(\left[AMD\right]\left(t\right)+\left[AM\right]\left(t\right)\right)\times \left(x_{1‐}x_{3}\right)\right)/\\ & \;\left(\left[AMDP\right]\left(t\right)+\left[{AMD}_{L}\right]\left(t\right)+\left[{AMD}_{H}\right]\left(t\right)+\left[AMD\right]\left(t\right)+\left[AM\right]\left(t\right)\right) \end{array}$$ (17)

Here the ordinary parentheses around *t*, indicate a functional dependence on *t* whereas the hook-parentheses indicate the probability of the respective state.

### 2.4 Fit of the Hill hyperbolic equation to data

Force-velocity data are fitted by the Hill (1938) hyperbolic equation (Hill, 1938) using non-linear regression (in Graph Pad Prism, v. 10.2.3, Graph Pad Software, LLC):

$$\left(F+a\right)\times \left(V+b\right)=b\;\left(Fo+a\right)$$ (18)

where *a* and *b* are constants, *F* is force at a given velocity, *V* is the velocity, and *Fo* is the maximal isometric force (at *V* = 0). The maximal velocity is given by: *V*_*0*_ = (*b* × *Fo*)/*a* because *V*_*0*_ occurs at *F* = 0.

## 3 Results

The model (Figure 1) is similar in structure to that previously used (Rahman et al., 2018; Månsson and Rassier, 2022) for simulation of skeletal muscle contraction but the parameter values (Tables 1, 2) are updated to be consistent with results from experiments on isolated β-myosin II molecules.

### 3.1 Adaptation of model to human cardiac muscle

We tested the model regarding its capacity to account for a range of ensemble contractile and kinetic properties of human ventricular muscle and actomyosin. This includes the maximum isometric force per cross-bridge, the maximum velocity of shortening, the actin-activated ATPase activity and the shape of the steady-state force-velocity relationship. We also tested the model against experimental single molecule mechanics data. Our strategy was to start with model parameter values from experiments on isolated proteins (Tables 1, 2, last column), primarily attributed to human β-myosin at 25°C (however, see notes of Tables 1, 2). We next modified these parameter values as guided by previous knowledge (e.g., (Rahman et al., 2018; Månsson and Rassier, 2022; Månsson, 2010)) and based on tested effects of the changes of the power-stroke, the force-velocity relationship and actin-activated ATPase.

**FIGURE 2:**
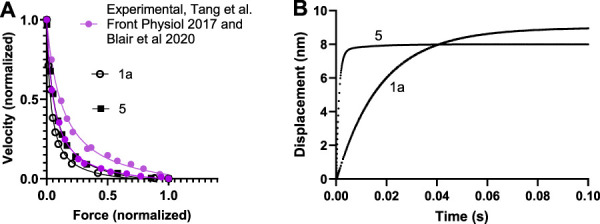
Force-velocity and power stroke data simulated for human cardiac muscle at 25°C using model in Figure 1 and parameter values in Tables 1, 2. **(A)** Force-velocity data simulated using parameter values in the third column of Tables 1, 2 (open black symbols; 1a in Supplementary Table S1) or optimized parameter values in 2nd column of Tables 1, 2 (filled black symbols, 5 in Supplementary Table S1). Simulations compared to experimental data of Tang et al. (Tang et al., 2016) (top) and Blair et al. (2020) (bottom). **(B)** Displacements during power-strokes simulated using parameter values in 3rd column of Tables 1, 2 (“1a”; see further Supplementary Table S1) or optimized parameter values in 2nd column of Tables 1, 2 (“5”, see further Supplementary Table S1) assuming stiff conditions (e.g., stiff optical trap; see *Materials and Methods*).

The parameter values from the literature (third column in Tables 1, 2) give reasonable simulated maximum isometric force (F_0_) per myosin head and a maximum velocity of shortening (V_0_) in the experimentally observed range as well as a steady-state ATP turnover rate close to 10 s^−1^ as found experimentally (Supplementary Table S1). The curvature of the force-velocity relationship is somewhat high (Figure 2A) corresponding to low maximal relative power output (W_0_; normalized to F_0_ x V_0_. However, particularly the rate constant of the displacement produced by the power-stroke in single molecule mechanics data is not well accounted for (Figure 2B). This could be amended by a simple increase in the rate constant k_LH-_ from 1,000 s^−1^ to 5,000 s^−1^ and a small increase of ΔG_LH_ from 10 to 12 k_B_T without other changes (Supplementary Table S1). However, direct use of the parameter values from experiments, whether k_LH-_ and ΔG_LH_ are increased or not, give lower maximum power-output than in experiments associated with a more curved force-velocity relationship (low a/F_0_^*^ in fits of the Hill (1938) equation (Equation 18; Supplementary Table S1; Supplementary Figure S1). Key parameter values were iteratively modified to overcome these limitations (Supplementary Table S1; Supplementary Figure S1) arriving at a set of parameter values (Tables 1, 2, second column) that give satisfactory fits to a range of experimental results (Supplementary Table S1; Figure 2; model 5). We use the parameter values in the second column in Tables 1, 2 in further simulations below unless otherwise stated. They give a power-stroke with similar half-time as observed experimentally. They also give values for the maximum velocity of shortening, curvature of the force-velocity relationship and the actin-activated ATP turnover rate consistent with experiments (Supplementary Table S1). However, the predicted maximum isometric force per cross-bridge is rather high compared to experimental values (see *Discussion*).

Using simulations with the parameter values in the 2nd column of Tables 1, 2 (ΔG_PiR_ = 5 k_B_T rather than 1 k_B_T) as starting points we performed a simple sensitivity analysis (Supplementary Figure S2), investigating the sensitivity of simulated features (force, velocity etc.) to different parameter values. In this analysis we made the simplifying assumption that there are no interactions between parameter values in their effects on the simulated properties. The main findings of the sensitivity analysis are that only a limited number of parameters (≤4) noticeably affect each of the simulated ensemble properties. A finding of interest is that the analysis suggests that a further reduced power-stroke distance would reduce the average isometric force per myosin head while reducing the curvature of the force-velocity relationship. This would make the simulated results better in line with experimental findings. However, we did not implement this change because it would make the parameter values for the first and second sub-stroke deviate too much from the experimental values from full length myosin (Tables 1, 2). For further details on the sensitivity analysis, please see the Supplementary Results.

### 3.2 Modelling OM effects

The simulations of the OM effects are performed under the assumption of 100% OM saturation and starting parameter values as described above (second column of Tables 1, 2 with ΔG_PiR_ = 5 k_B_T). We further assume full activation and that the two myosin heads work independently of each other. These assumptions are discussed below. Finally, we assume in the modelling that OM binds to myosin in the detached pre-power-stroke state (MDP in Figure 1) and immediately dissociates from myosin if it undergoes the main force-generating transition (AMD_L_ - > AMD_H_) (Planelles-Herrero et al., 2017).

We considered the operation of one or several molecular mechanisms of action of OM implied by single molecule mechanics and solution kinetics studies. The key hypotheses for the OM effects are thus: 1. inhibition of the power-stroke (Planelles-Herrero et al., 2017; Rohde et al., 2017; Liu et al., 2018; Woody et al., 2018; Auguin et al., 2024) (corresponding to reduction of k_LH-_ and/or reduced difference in affinity between the AMD_L_ and AMD_H_ states (reduced ΔG_LH_), 2. Increased equilibrium constant for the hydrolysis reaction (Liu et al., 2015; Rohde et al., 2017), 3. Reduced Pi-affinity in the presence of OM (Governali et al., 2020). 4. Increased actomyosin affinity in pre-power-stroke states (Swenson et al., 2017) corresponding to increases in ΔG_on_ and ΔG_PiR_ and finally 5. Shift of the optimal attachment position of myosin from the minimum of the elastic free energy to a position with increased elastic free energy corresponding to force in the muscle shortening direction (Auguin et al., 2024). Point 4 is consistent with increased sliding velocity with increased ionic strength in the presence of OM considering that pre-power-stroke states are believed to be dominated by electrostatic interactions. Point 5 is suggested by modelling based on molecular structures showing that the lever arm is more primed in the pre-power-stroke state in the presence of OM (Auguin et al., 2024).

For modelling the OM-effects on the power-stroke, we assumed reductions in the parameters ΔG_LH_ and k_LH-_ as justified in detail, in the Discussion. A substantial decrease in k_LH-_ without any change in ΔG_LH_ predicts some effects of OM in directions seen in experiments. This includes a reduction of the maximum shortening velocity with only minor changes of isometric force (Supplementary Figure S4A). It also includes an inhibition of the power-stroke (Supplementary Figure S4B), a small increase in the number of attached cross-bridges during isometric contraction but no changes in the actin-activated ATPase rate (inset Supplementary Figure S4A). A substantial reduction in ΔG_LH_ without any change in k_LH-_ reduces both maximum velocity and maximum isometric force while the power-stroke is inhibited as with an isolated reduction in k_LH-_ (Supplementary Figures S4C, D). Moreover, there is a small increase in the number of attached cross-bridges during isometric contraction but no change in the actin-activated ATPase.

**FIGURE 3:**
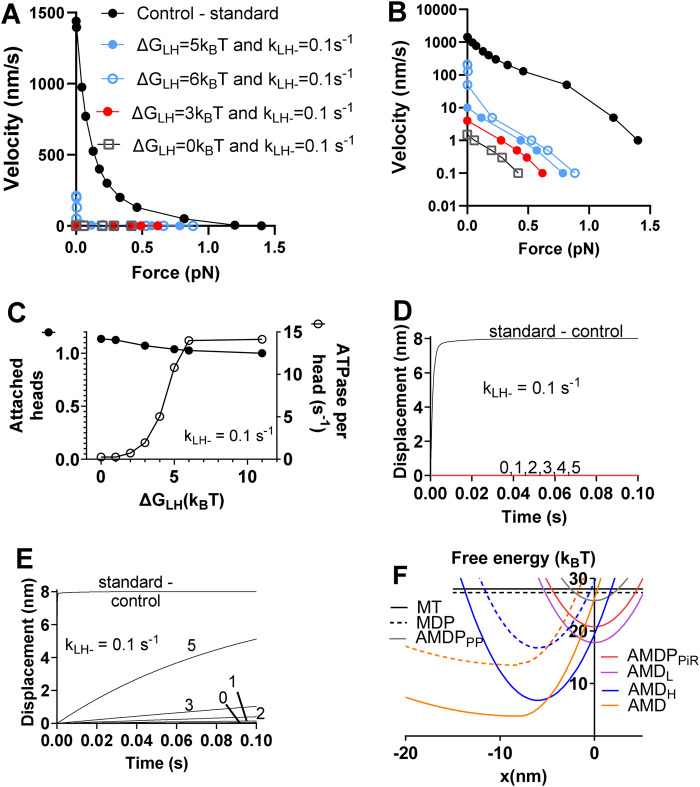
Simulation of effects on the force-velocity relationship, the power-stroke and the actin-activated ATPase of combined reductions in ΔG_LH_ and k_LH-_ without changes in other parameter values. **(A)** Force-velocity relationship. **(B)** Force-velocity relationship as in A but with a logarithmic scale for the velocity axis to more clearly visualize the low-velocity range. **(C)** Number of attached myosin heads in isometric contraction (filled symbols, left vertical axis) normalized to the control value and actin activated ATPase (open symbols, right vertical axis) vs. ΔG_LH_ for a constant value of k_LH-_ = 0.1 s^−1^. **(D)** Effects on displacement associated with power-stroke of reduction in ΔG_LH_ from control value of 11 k_B_T down to 0 k_B_T and as indicated by numbers in figure with k_LH-_ = 0.1 s^−1^. Stiff optical trap (>>3 pN/nm) assumed. **(E)** Effects on displacement associated with power-stroke of reduction in ΔG_LH_ from control value of 11 k_B_T down to 0 k_B_T as indicated by numbers in figure with k_LH-_ = 0.1 s^−1^. Soft optical trap (0.07 pN/nm) assumed. **(F)** Free energy diagrams from Figure 1 with the effects of OM in the simulations indicated by dashed lines.

Next, considering the limited effects of separate reductions in ΔG_LH_ and k_LH-_ (Supplementary Figure S4), we evaluated the effects of combined reductions in the values of these parameters (Figure 3). After testing different combinations of such changes (Figure 3A) we found that a simultaneous reduction in ΔG_LH_ from 11 to the range 0–3 k_B_T and k_LH-_ from 5,000 to 0.1 s^−1^ leads to >100-fold reduction of the maximum velocity of shortening but a reduction of the maximum isometric force by only 60%–80% (Figures 3A, B; Table 3). The number of attached cross-bridges in isometric contraction under these simulated conditions is slightly increased compared to control conditions. The actin-activated ATPase, on the other hand, is reduced almost 100-fold (Figure 3C). Finally, the power-stroke, investigated under stiff trapping conditions (as in Supplementary Figure S1 and corresponding to the situation in muscle cells) is inhibited (Figure 3D). We also (Figure 3E) simulated soft trap conditions (trap stiffness 0.07 pN/nm) as used in (Woody et al., 2018) by changing the free energy diagrams (otherwise shown in Figure 3F) as indicated in Supplementary Figure S5. As expected, this predicts a faster stroke in the absence of OM (approaching the conditions with actomyosin in solution without any elastic constraints). Moreover, the simulated power stroke rates are less reduced for each combination of reductions of ΔG_LH_ and k_LH-_ under soft trap conditions. If fixing the reduction in k_LH-_ from 5,000 to 0.1 s^−1^, simulation of the effect on the power stroke observed in (Woody et al., 2018) required a reduction of ΔG_LH_ from 11 to 1 k_B_T or lower (Figure 3E).

**TABLE 3:** Reproduction of experimentally observed OM effects on actomyosin kinetics, motility and force-development by different molecular mechanisms.

OM model version	Model parameters changed	Change in isometric force	Change in isometric fraction attached heads	Change in max velocity	Change in ATPase	Change in power-stroke amplitude <100 ms
O1	ΔG_LH-_ reduced from11 k_B_T to 1 k_B_T	−52.2%	+7.2%	−42.0%	0%	−99% (0%)^a^
O2	k_LH-_ reduced from 5,000 s^−1^ to 0.1 s^−1^	−5.1%	+2.4%	−39.4%	0%	−100% (0%)^a^
O3	O1 + O2	−68.1%	+15.8%	−99.9%	−98.1%	−100% (0%)^a^
O4	O3 + K_3_ increased from 2 to 6	−66.2%	+15.8%	−99.8%	−98.1%	−100% (−98.1%)^a^
O5	O4+Kc increased from 10 to 100 mM	−58.6%	+21.0%	−99.9%	−98.1%	−100% (−98.1%)^a^
O6	O5 + ΔG_on_ increased from 1.5 to 4 k_B_T	−36.9%	+40.4%	−98.8%	−98.1%	−100% (−98.1%)^a^
O7	O6 + ΔG_PiR_ increased from 5 to 5.5 k_B_T^b^	−34.4%	+42.1%	−98.8%	−98.1%	−100% (−98.1%)^a^
O8	O7+ε = 1 nm	−1.9%	+49.1%	−95.6%	−98.1%	−100% (>-98.1%)^a^
O9	As O7 but compared to control with ΔG_PiR_ = 1	−20.8%	+62.0%	−98.7%	−98.1%	−100% (−98.1%)^a^
O10	As O8 but compared to control wi ΔG_PiR_ = 1	+18.5%	+70.0%	−95.5%	−98.1%	−100% (>-98.1%)^a^
O11^c^	As O10 but with detachment from ADP_L_ state k_ADP_o = 1 s^−1^ or (4 s^−1^)	−8.4% (−32.3%)	+36.0% (+18.9%)	−95.5% (−94.9%)	−91.0% (−69.8%)	−100% [−98.1%, (−97.6%)]^a^
OM effects in experiments	Not applicable	Un-changed or small increase (Governali et al., 2020; Scellini et al., 2024)	Un-changed (Governali et al., 2020)	20 - >100–fold reduced (Swenson et al., 2017; Liu et al., 2018; Woody et al., 2018)	4.5-fold reduced (Swenson et al., 2017)	Completely inhibited (Woody et al., 2018)

^a^ “Soft” trap simulation (stiffness 0.07 pN/nm) in parenthesis.

^b^ Not bigger change possible because of instabilities in numerical computations.

^c^ Most faithful reproduction of experimental data.

In summary, the combined reduction in ΔG_LH_ from 11 to 1 k_B_T and k_LH-_ from 5,000 to 0.1 s^−1^ accounts qualitatively for all OM effects and quantitatively for all effects except that on the actin-activated ATPase and that on the maximum isometric force. The effect on the ATPase is much more prominent than in experiments. As proposed previously (Woody et al., 2018), this can be explained by slow “escape” detachment from the pre-power-stroke state (AMD_L_ in present model) with subsequent completion of the ATP turnover. Regarding the isometric force, this is in most studies somewhat increased by OM (Malik et al., 2011; Aksel et al., 2015; Scellini et al., 2024) (although with exceptions (Governali et al., 2020)). In our continued studies to elucidate this effect, we assume that OM reduces ΔG_LH_ from 11 to 1 k_B_T and k_LH-_ from 5,000 to 0.1 s^−1^ while also considering additional mechanisms.

An effect of OM that has been convincingly demonstrated in more than one study is a shift of the hydrolysis equilibrium towards the post-hydrolysis MDP state (increase in K_3_) by a reduction in the rate of the backwards transition (Liu et al., 2015; Rohde et al., 2017). However, this additional change in the model, only slightly increased the maximum isometric force (Table 3) without other effects.

While now keeping the above assumed OM-induced changes in both k_LH-_, ΔG_LH_ and K_3_, we next also reduced the Pi-affinity by increasing Kc from 10 mM to 100 mM. This goes in the same direction as suggested by effects of OM on the isometric force at varied Pi in slow skeletal muscle fibers (Governali et al., 2020). An increase of Kc from 10 mM to 100 mM attenuates the reduction in the isometric force in the model from about 70% without the mechanism to about 60% (Table 3). No other simulated OM effects were changed by inclusion of this mechanism.

We next assumed the additional effects of increased affinity between actin and myosin in pre-power-stroke states reflected in increases of ΔG_on_ and ΔG_PiR._ To illustrate the effect of changes in these parameters we tentatively increased ΔG_on_ from 1.5 to 4 k_B_T and ΔG_PiR_ from 5 to 5.5 k_B_T. These changes together (Table 3), further attenuate the reduction in the isometric force in simulation of the OM data, with minimal other effects.

**FIGURE 4:**
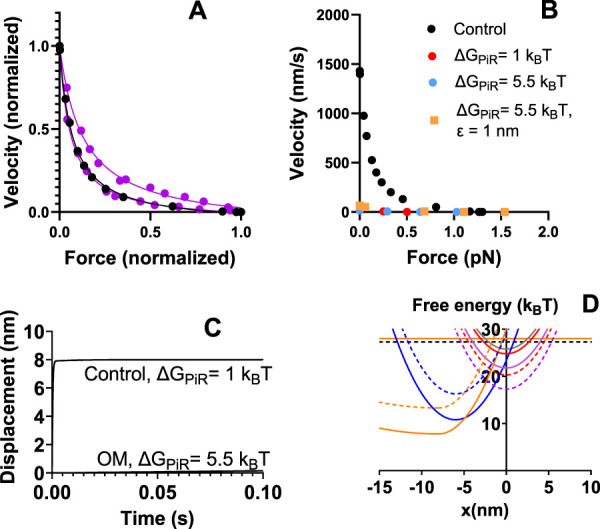
Model with reduced value of ΔG_PiR_ compared to Figure 3. **(A)** Simulation (black) of control force-velocity data assuming ΔG_PiR_ = 1 k_B_T instead of ΔG_PiR_ = 5 k_B_T. The parameter values used are otherwise given in the second column of Tables 1, 2. Simulations by solution of differential equations in state probabilities (filled circles) compared to the same experimental data (Tang et al., 2016; Blair et al., 2020) as reproduced in Figure 2. **(B)** Simulation of OM effects on the force-velocity relationship compared to control data (black) reproduced from panel **(A)**. In all simulations of the OM effects (red, blue, orange) the following changes from the second column in Tables 1, 2 are assumed: ΔG_LH_ = 1 k_B_T, k_LH-_ = 0.1 s^−1^, Kc = 100 mM, ΔG_on_ = 4 k_B_T. For red data, ΔG_PiR_ = 1 k_B_T. For blue and orange data ΔG_PiR_ = 5.5 k_B_T. In addition, ε = 1 nm for orange data but ε = 0 nm for all other simulations. **(C)** Simulation of OM effects on the displacement associated with the power-stroke under conditions similar to those of orange and blue data points in B with control data simulated on the assumption that ΔG_PiR_ = 1 k_B_T and all other parameter values as in Tables 1, 2 (2nd column). Simulation of power-stroke assuming soft optical trap (0.07 pN/nm). **(D)** Free energy diagrams from Figure 1 modified to assume ΔG_PiR_ = 1 k_B_T for control conditions. Free energy diagrams for OM conditions as indicated by dashed lines.

It would be of interest to investigate if a further increase in ΔG_PiR_ would give even better quantitative agreement with the experimental findings that OM increases, rather than decreases the isometric force. This was prevented in the analysis above by numerical instabilities with a high value of ΔG_PiR_. We noted above that the simulated isometric force under control conditions is somewhat higher than in experiments while the maximum relative power output is lower. Interestingly, we noted in pilot simulations that a substantial, several-fold, reduction in ΔG_PiR_ reduces the maximum force while increasing the relative maximum power. We therefore simulated control data after changing ΔG_PiR_ from 5 to 1 k_B_T. This led to results with satisfactory fit to experimental force-velocity data (Figure 4A), similar actin-activated ATPase as with ΔG_PiR_ = 5 k_B_T, somewhat reduced average isometric force (from 1.4 to 1.26 pN/head) and similar power-stroke rate.

In view of the success of reducing ΔG_PiR_ under control conditions to 1 k_B_T, we now compared simulated OM effects to control conditions with ΔG_PiR_ = 1 k_B_T. First, we simulated the OM effects with ΔG_LH_ = 1 k_B_T, k_LH-_ = 0.1 s^−1^, K_3_ = 6, Kc = 100 mM and ΔG_on_ = 4 k_B_T. The simulated force-velocity data and the power stroke under soft trap conditions are shown in Figures 4B, C. By further assuming increased ΔG_PiR_ from 1 to 5.5 k_B_T in the presence of OM (Figures 4B–D) we found attenuated OM-induced reduction in isometric force (see also Table 3, O7 vs. O9). Finally, we tested whether it is possible within the framework of the model and proposed molecular mechanisms of OM to reproduce an increased isometric force in the presence of the drug while still accounting for other contractile effects. To this end we assumed that the average strain of the myosin cross-bridges is higher at the point of attachment as suggested by the “overpriming” of the lever arm in the pre-power-stroke state in the presence of OM (Auguin et al., 2024). This was implemented by simply shifting the peak of the Gaussian function in x for the attachment probability away from the minimum of the free energy profile by a quantity ε = 1 nm. We are not entirely certain about the optimal approach for implementing this mechanism. However, it is shown in Figures 4B, C; Table 3 (O9) that the change in ε from 0 to 1 nm, in addition to the above changes in parameter values, increase the isometric force. The other effects of OM such as reduction of ATPase rate, power-stroke rate, increase in number of cross-bridges during isometric contraction and reduction in velocity are all accounted for [Table 3 (O9) and Figures 4B, C]. The reduction in the maximum actin-activated ATPase (nearly 100%) is substantially larger than the 4.5 – fold reduction seen in experiments (Swenson et al., 2017). The remaining rather high ATP turnover in the experiments at saturating OM can be attributed to non-conventional paths suggested previously (Liu et al., 2015; Swenson et al., 2017; Liu et al., 2018; Woody et al., 2018). One possibility that would account for a less substantial reduction in the ATPase with expected lower effect on other simulated properties would be a slow (about 10 s^−1^ in previous work) escape route with cross-bridge detachment from the AMD_L_ state (Woody et al., 2018). The effects of incorporating such a transition (this time with rate constant either 1 or 4 s^−1^) are given in Table 3 (O11).

## 4 Discussion

### 4.1 Summary and relevance of the results

We have adapted and validated a model previously used to simulate skeletal muscle contraction (Rahman et al., 2018; Månsson and Rassier, 2022) for simulation of human cardiac actomyosin operation. We assume full activation. Accordingly, we do not consider regulatory roles of accessory proteins such as troponin, tropomyosin, myosin-binding protein C, titin etc. Neither do we consider related thick filament mechano-sensing based activation, relying on different degrees of parking of the myosin heads on the thick filament backbone in an interacting heads motif (Brunello and Fusi, 2024). The model is therefore not directly useful for simulation of cardiac contraction *in situ* where the omitted mechanisms play critical roles during the cardiac cycle (Brunello and Fusi, 2024). The model is instead designed for evaluating key aspects of the actin-myosin based force-generation mechanism *per se*. This includes basic energy transduction mechanisms as well as effects of mutations and small-molecular myosin-active substances with particular emphasis on how molecular mechanisms translate into effects on the ensemble level. Because of the mentioned limitations, the model predictions would ideally be tested using reconstituted pure actin-myosin experimental systems (Liu et al., 2024) from single molecule to ensemble levels e.g., (Pertici et al., 2018; Cheng et al., 2020; Hwang et al., 2021). We demonstrate the usefulness of the model in elucidating effects of OM and we expect it to be useful for studying other cardioactive drugs e.g., mavacamten and afficamten. Despite not considering effects of interacting head motifs and superrelaxed states believed to be important in drug mechanisms *in situ* we argue that the model is still relevant in this regard. This follows from recent findings (Chu et al., 2021; Mohran et al., 2024) that can be interpreted to play down the importance of inter-head interactions in the drug effects in favor of mechanisms on the single head level.

### 4.2 General model characteristics in relation to other work

The detailed mechanokinetic model for cardiac ventricular muscle has the same states and transitions as the previous models for skeletal muscle from which it is developed. The model is updated with transition rate constants from studies of isolated human cardiac myosin. Generally, these rate constants, except those believed to be attributed to simple diffusion (e.g., the actual Pi-release step) or structural transitions in the motor (power stroke) are lower than in previous skeletal muscle models consistent with rather slow cardiac contraction. The attachment rate constant (k_on_´ΔG_on_) is appreciably lower than for fast skeletal muscle which accounts for slower actin-activated ATPase, lower Pi-release rate (limited by attachment rate (Stehle, 2017)) and slow rate of rise of isometric force. The ADP release rate constant is also appreciably slower (about 100 s^−1^) (Swenson et al., 2017; Bodt et al., 2024) than in fast skeletal muscle (>1,000 s^−1^) (Nyitrai et al., 2006). Effective strain-dependence for the ADP-release is achieved by a strain-dependent fast equilibrium prior to ADP release in similarity to previous use of the model for skeletal muscle. This is important (Huxley, 1957; Duke, 1999) to adapt the muscle to varying loads.

We did not assume the ATP-induced detachment to be strain dependent as in several previous models for skeletal muscle [e.g., (Månsson and Rassier, 2022)]. Instead, we accounted for a relatively high maximum sliding velocity as in some earlier studies (Huxley and Tideswell, 1996; Kaya et al., 2017; Månsson et al., 2019; Hwang et al., 2021) by assuming non-linear cross-bridge elasticity with low stiffness of the cross-bridges in the AM-, and AMD-states at negative x. Although the presence of such non-linearity is controversial (Linari et al., 2020) it was already noted by Huxley and Tideswell (Huxley and Tideswell, 1996) that either non-linear elasticity as used here, or strain-dependent ATP-induced detachment rate is necessary to account for the high velocities observed. One may also consider the possibility that the strain dependence of different transitions differs between cardiac and skeletal muscle myosin II isoforms (Mijailovich et al., 2017).

Most previous models with detailed description of the mechanokinetic cycle have focused on skeletal muscle. Models of cardiac contraction have often had different aspects of the activation mechanism in focus (Rice et al., 2008; Campbell et al., 2010). More recent efforts (Campbell et al., 2020; Sharifi et al., 2024; Kimmig and Caruel, 2020; Tomasevic et al., 2023) have aimed towards complete models that account for cardiac contraction on different scales from single molecules up to the organ and even organism level while also integrating key regulatory (activation) mechanisms. Whereas some studies only cover lower organizational scales, inclusion of regulatory mechanisms have generally been central [e.g., (Fenwick et al., 2021; Mijailovich et al., 2021; Kosta et al., 2022)]. The mentioned developments are commendable for organ and organism physiology as well as clinical purposes [e.g., (Rodero et al., 2023)], because in their normal function, cardiac muscle cells operate under continuously varied activation levels of both the thin and thick filaments. Models aiming to cover all diverse aspects of cardiac contraction, often treat the actin-myosin interaction mechanism in a less detailed way (e.g., to limit computational cost) thereby also reducing the potential to decipher details of the energy transduction mechanisms and the effects of drugs on this mechanism. However, within some large-scale modelling projects rather detailed studies of the actin-myosin interaction have been performed, focusing on the effects of cardiomyopathy mutations (Ujfalusi et al., 2018; Vera et al., 2019). Whereas some considerations related to effects of load on the kinetics were considered in these papers detailed force-velocity data and myosin power-strokes were not simulated. Also, some other models that include a wide array of regulatory mechanisms include detailed reproduction of the actin-myosin interaction. However, the main aim of such models [e.g., (Mijailovich et al., 2021)] are often to better reproduce the cardiac contraction rather than study details of the actin-myosin interaction mechanism for more in-depth insight into energy transduction or drug mechanism. The model used here has most in common with that in (Hwang et al., 2021). However, not all model states of (Hwang et al., 2021) are directly analogous to those assumed here as some of these states are inspired by previous studies of drug effects in skeletal muscle (Rahman et al., 2018).

### 4.3 Model of OM effects and implications for energy transduction

**FIGURE 5:**
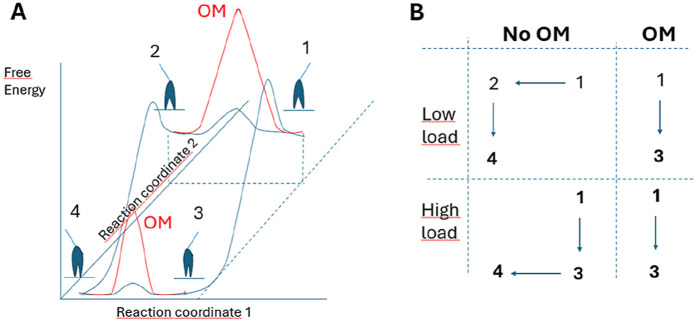
Model allowing either lever-arm swing or 50 kD cleft closure to occur first in myosin force-generation. **(A)** Schematic (tentative) activation energy diagram for lever arm swing and 50 kD cleft-closure in the absence of load. The diagram in the presence (red and blue) and absence (blue) of OM assumes that OM primarily increases the activation energy for the lever arm swing. **(B)** Likely path between states 1–4 in the model in A under conditions of high and low load in the absence and presence of OM. This means that a substantial reduction of k_LH-_ (corresponding to increased activation energy for the lever arm swing) would make the path 1 to 3 to dominate in the presence of OM even in the absence of load, corresponding to a lower average free energy in the pre-power-stroke state (increased Kc-value) together with reduced value of ΔG_LH_.

Strikingly, most effects of OM are reasonably well accounted for (without other changes), even quantitatively, by appreciable reductions in the values of the parameters k_LH-_ and ΔG_LH_ that govern the power-stroke kinetics. The power stroke (lever arm swing), that follows Pi-release from the active site in the model, is coupled to increased actin-affinity due to closing of the 50kD myosin cleft, possibly with the two structural events occurring almost simultaneously (Robert-Paganin et al., 2020). However, it is of interest to consider the possibility that either the swing or cleft closure comes first (Malnasi-Csizmadia and Kovacs, 2010; Caremani et al., 2013; Rohde et al., 2017) (Figure 5) but with the pre-power-stroke and post-power-stroke lever arm positions most stable with open and closed cleft, respectively. Without hindrance by counteracting tension, e.g., solution studies with actomyosin, the lever arm may swing first, followed by cleft-closure with increased actin-affinity to stabilize the post-power-stroke lever arm position. Under such conditions unhindered thermal fluctuations are expected to be very fast (in the ns range) (Howard, 2001). If the lever arm swing is indeed unhindered it seems likely that it is faster than movement of the cleft-region as the latter may be limited by internal constraints in the motor domain of myosin and/or molecular friction at the actin-binding interface. On the other hand, under counteracting load, it seems reasonable that the actin-affinity should increase before the power-stroke to be able to sustain force-development. This argues for a power-stroke after cleft-closure in muscle contraction, when there is hindering load. The increase in actin-affinity upon cleft-closure would then provide a free energy gradient to overcome the elastic forces that resist the swing towards the post-power-stroke position (Eisenberg and Hill, 1978; Hwang and Karplus, 2019).

In terms of this dual-path view, we propose that a primary effect of OM, as suggested by several studies (Planelles-Herrero et al., 2017; Rohde et al., 2017; Woody et al., 2018; Auguin et al., 2024) is a greatly increased activation energy for the lever-arm swing (Figure 5A) after Pi-release. This effect would correspond to great reduction of the rate constant k_LH+_(x) in the model. In practice, on the molecular level, it would mean that the drug essentially “glues“ the converter domain and the connected lever arm to the upper 50 kD domain of the myosin head as suggested in (Auguin et al., 2024) [see also (McMillan et al., 2025)]. The increased activation energy for the lever-arm swing would effectively prevent it even in solution without resisting load. It is then possible that the lever arm swing is delayed behind the cleft closure. Because the hindrance of the lever arm swing is likely to remain effective even after the increase in affinity, OM may promote high-affinity states with the lever arm in a pre-power-stroke position (Figures 5A, B). This is consistent with the reduction in ΔG_LH_. However, interestingly, the idea of high-affinity states with the lever arm in pre-power-stroke positions also accords with other mechanisms included in the more advanced versions of the model (Table 3). This includes the increase in ΔG_on_, ΔG_PiR_ and Kc reflecting increased actin affinity in the pre-power-stroke states AMDP_PP,_ AMDP_PiR_ and AMD_L_.

**FIGURE 6:**
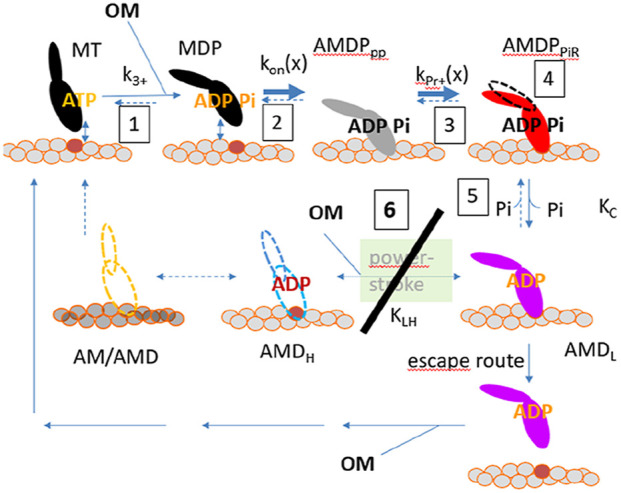
Molecular mechanisms of action of OM. All mechanisms considered above are numbered from 1 to 6 starting in the upper left corner. OM binding occurs to the MDP state and is assumed to leave its binding pocket upon completion of the power-stroke or after detachment via an “escape” route. The OM mechanisms are: 1. Shift of the hydrolysis equilibrium, 2. Increased attachment rate, 3. Increased affinity in the Pi-release state (AMDP_PiR_), 4. Overpriming of the lever arm in a pre-power-stroke, 5. Reduced Pi-affinity, 6 (major mechanism): almost complete inhibition of power-stroke by reduction of k_LH-_ and ΔG_LH_ in the model. The escape route is likely to exist also in the absence of OM but would not be relevant when the power-stroke is very fast.

The increase in Kc is also consistent with effects of OM on the Pi-sensitivity of isometric force in slow skeletal muscle (Governali et al., 2020). Moreover, there has been evidence from *in vitro* motility assays at varied ionic strength that OM increases the actin-affinity of weakly bound pre-power-stroke states (Swenson et al., 2017). This would be expected to include the AMDP_PP_ state and the AMDP_PiR_ state for which ionic interactions between actin and myosin are likely to be important (Llinas et al., 2015). These mechanisms are consistent with the increases in both ΔG_PiR_ and ΔG_on_ proposed above. The latter effect is also consistent with increased actin affinity (lower K_app_) and saturation of the actin-activated ATPase at lower actin concentration in the presence of OM (Swenson et al., 2017). The shift of the rate limiting step of the actin-activated ATPase cycle from the attachment step to the lever arm swing with myosin attached to actin would contribute to reduced K_app_. The molecular mechanisms discussed above are illustrated schematically in Figure 6. The mechanisms shown there are quite similar to those proposed in (Woody et al., 2018) but with more details.

OM as well as other small molecular myosin modulators such as MAVA have been proposed to exert indirect effects on activation of the thin and thick filaments in addition to the interaction between individual myosin motor domains and actin. This includes enhanced cooperative activation of the thin filaments by altered degree and duration of myosin binding to actin as well as reduced fraction of the interacting head motif of the two myosin motor domains of each myosin molecule (Kampourakis et al., 2018; Woody et al., 2018). However, these complexities are secondary to the direct effects of the small molecular compounds on each motor domain considered here. Moreover, recent studies of MAVA indicate that the direct effects on the myosin motor domain may have more relevance in the clinically important effects of the drugs than previously believed (Chu et al., 2021; Mohran et al., 2024).

Previous modelling of the OM effects have used rather simple models of the actin-myosin interactions (Rohde et al., 2017; Woody et al., 2018; Governali et al., 2020). Governali and co-workers (Governali et al., 2020) interpret their data from studies of OM effects on isometric contraction of slow rabbit skeletal muscle fibers in terms of a branched kinetic scheme. Some key features to their interpretation are consistent with the present model such as inhibition of the power-stroke and increased affinity in pre-power-stroke states. Additionally, an escape pathway with detachment from actin from a pre-power-stroke was considered. Other authors (Rohde et al., 2017), also use a branched kinetic scheme to interpret their transient kinetics solution data for Pi-release and the power-stroke. Based on their experimental findings they propose a model reminiscent of our Figure 5 in structure. However, unlike our view where we assume that the order between lever-arm swing and cleft-closure is reversed by OM, Rohde et al. (2017) instead interpret their data to mean that the order between Pi-release from the active site and the power-stroke is reversed by OM. In our model, the Pi-release always precedes the power-stroke [see also (Planelles-Herrero et al., 2017)] whether OM is present or not. Whereas, the interpretation of (Rohde et al., 2017), seems consistent with their data there are alternative explanations if a secondary Pi-binding site outside the active site is considered (Llinas et al., 2015; Planelles-Herrero et al., 2017; Moretto et al., 2022). The latter multistep Pi-release model also accounts for a broader range of data in the absence of OM than the idea with a power-stroke before Pi-release (Moretto et al., 2022).

Kinetic schemes like those in (Governali et al., 2020; Rohde et al., 2017) do not explicitly consider the strain and associated elastic energy of the model states, preventing their use in conditions with varying cross-bridge strain, e.g., length steps and muscle shortening. Also, one given set of rate constants cannot account for both actin-activated myosin ATPase in solution and isometric contraction because several rate constants are strain-sensitive. Finally, not even an apparently static steady-state condition such as isometric contraction may be unambiguously interpreted in terms of model states in a kinetic scheme. The reason is that changes in the average population of each given cross-bridge state and average inter-state transition rate constants do not necessarily reflect the key effect of a molecular change. The latter may, instead, cause changes in the spatial distribution and range of cross-bridge strains in each state with minimally changed average values (Månsson et al., 2023). In contrast to the kinetic schemes, full mechanokinetic models that explicitly consider a range of elastic strains and forces, can account for all diverse contractile phenomena using one given set of parameter values (Rassier and Månsson, 2025). Neither Governali et al. (2020) nor Rohde et al. (2017) explicitly consider such a full range of phenomena due to the mentioned limitations. It is difficult to assess how well an extended version of their schemes, with a range of strains for each state, would quantitatively account for the full range of OM effects investigated here.

Woody et al. (2018) performed Monte-Carlo simulations with 75 myosin heads interacting with an actin filament or a regulated thin filament assuming a two-state cross-bridge model and a simple cooperative activation model. After attachment, a power-stroke rapidly occurred followed by detachment of the cross-bridges. The authors could account for all their ensemble results as well as previous results (Nagy et al., 2015) for isometric force at varied pCa by an inhibited power-stroke and an increased rate of cross-bridge attachment in the presence of OM. Thus, their key results are broadly consistent with the present modelling results albeit with less details regarding molecular mechanisms.

### 4.4 Limitations

We have already considered specific limitations above in that we assume full activation level and do not consider possible inter-head interactions. However, there are also general challenges relevant to most modelling efforts. First, it is difficult to obtain all model parameter values under coherent conditions regarding myosin isoform, species, temperature, ionic strength, etc. Second, it is challenging to compare model predictions of ensemble behavior to experimental data (often from muscle fibers, myofibrils etc.) obtained under similar conditions as those used to derive model parameter values.

We have aimed to use experiments on human cardiac ventricular myosin heavy chains both for deriving parameter values and ensemble contractile properties against which the model predictions are tested. However, some parameter values partly rely on bovine and porcine ventricular β-myosin (see notes of Tables 1, 2). Moreover, most of the data on isolated proteins rely on expressed β-myosin heavy chains with regulatory and essential light chains from mouse skeletal muscle (expression host). Whereas some studies (Deacon et al., 2012; Tang et al., 2021) have shown only minor mechanokinetic effects of the light chain composition, other studies have found quite substantial effects (Nayak et al., 2020; Osten et al., 2022; Wang et al., 2022) which is important to bear in mind.

A discrepancy that we note for the control conditions is that the model predicts a higher value of the average isometric force per myosin head than seen in experiments. This is particularly striking if the real situation in a muscle involves three sites per 36 nm repeat (three-site model) along the actin filament instead of 1 (one-site model) as assumed in the present modelling. In the one-site model the average force per head (whether attached or not) with optimal parameter values is 1.3 pN. If the three-site model is correct, the latter value should be multiplied by 3 giving 3.9 pN per head. This is appreciably higher than estimated from experimental data for isometric force at 25°C (Supplementary Table S1) which suggest 1-2 pN per head. The model predicts fractions of strongly attached heads of 18% and 54% for the one-site and three-site model, respectively and a force per attached head of 7 pN. The discrepancies regarding maximum isometric force may be partly attributed to general limitations and uncertainties considered above. However, there are also other possible mechanisms that may account for this effect as considered in a Supplementary Discussion.

In view of the uncertainties discussed above it is not meaningful to optimize the fits to experimental data using quantitative methods. Instead, we rely on a bottom-up approach where we start from independently obtained parameter values and modify these as little as possible. It should be noted in this context that the parameter values which we finally use (2nd column, Tables 1, 2) only differ minimally from those suggested by literature data (3rd column, Tables 1, 2).

A limitation in the studies of OM is that we only consider effects of saturating OM concentrations. Moreover, we do not consider the evidence for two exponential processes found using both transient biochemical kinetics (Liu et al., 2015; Rohde et al., 2017; Swenson et al., 2017) and optical tweezers based single molecule mechanics (Liu et al., 2018; Woody et al., 2018). Different origins of the two processes have been considered. It seems that even at saturating OM concentration, a fraction of the myosin molecules with OM continue along a path only slightly modified from the normal cycle whereas a dominant fraction pass through a greatly modified cycle with much slowed power-stroke (Liu et al., 2018). The latter cycle is in focus here and we assume that the fraction moving through the normal cycle is negligible. However, we briefly consider the effect of possible “escape” detachment from the AMD_L_ state suggested by (Woody et al., 2018).

## 5 Conclusions and perspectives

We have shown that a model previously used to simulate skeletal muscle contraction at full activation can account also for cardiac ventricular contraction after appropriate updates of the model parameter values. Moreover, the model previously used for insight into drug effects on skeletal muscle, also accounts for contractile effects of the cardiotonic substance omecamtiv mecarbil. Particularly, the modelling suggests that most OM actions at full activation are primarily ascribed to the inhibition of the power-stroke. However, the modelling studies are also consistent with the operation of several other drug-related mechanisms proposed in previous work and which we show are logically connected. The usefulness of the modelling in elucidating the OM effects, together with similar previous use with skeletal muscle, suggest that similar modelling would be of value to elucidate the detailed mechanisms of other cardioactive drugs. This includes mavacamten, afficamten and danicamtiv (Lehman et al., 2022; Spudich, 2024) but could also be applied to newly discovered substances.

## Data Availability

The original contributions presented in the study are included in the article/Supplementary Material, further inquiries can be directed to the corresponding author.
