# Supplementary material for: Mechanistic insights into effects of the cardiac myosin activator omecamtiv mecarbil from mechanokinetic modelling
Source: Front Physiol. 2025 Apr 17;16:1576245. doi: 10.3389/fphys.2025.1576245 (PMC12043640; doi:10.3389/fphys.2025.1576245)
Supplement: Supplementary file 1 [file DataSheet1.pdf]

Supplementary data

Mechanistic insights into effects of the cardiac myosin activator omecamtiv mecarbil from mechanokinetic modelling

by

Alf Månsson

Supplementary Methods

The attachment step of myosin cross-bridges into the $AMDP_{PP}$ state would be expected to occur from a non-specifically weakly bound state $AMDP_W$ in rapid equilibrium with the MDP state, rather than directly from the MDP state. This rapid equilibrium is governed by Equation S1:

$$K_w = \frac{[AMDP_w]}{[MDP][A]} \qquad \text{(S1)}$$

where $[AMDP_w]$, [MDP], [A] is the concentration of the species $AMDP_w$, MDP and actin, respectively. In experiments with muscle fibers the actin concentration is fixed at an effective concentration $[A]_{eff}$. This simplifies equation S1 to Equation S2:

$$K_w^{const} = \frac{[AMDP_w]}{[MDP][A]_{eff}} \qquad \text{(S2)}$$

The cross-bridge attachment rate from the $AMDP_w$ state would be given by:

$k_{on}''\,[AMDP_w] = k_{on}''\,K_w^{const}[MDP][A]_{eff}$

where $k_{on}' = k_{on}''\,K_w^{const}\,[A]_{eff}$ is used in Eq. 1 in the main paper.

Supplementary Results

Iterative fine-tuning of model parameters for control conditions

Key parameter values were modified to overcome limitations in reproducing force-velocity data and displacements during the power-stroke when using parameter values derived directly from the literature. Particularly, a change in x_2 from -6 to -7 nm with x_3 kept constant at -9 nm increased the maximum relative power output, W_0. This change corresponds to a change in the distribution of the power-stroke between two sub-strokes so that the first sub-stroke is longer whereas the second sub-stroke is shorter. To increase the power output further we also increased the attachment rate constant by increasing ΔG_{on} from 1 to 1.5 k_BT. Additionally, W_0 was further increased by a reduction in the ADP dissociation rate constant k_6 from 100 to 75 s^{-1} which, however, also reduced the maximum velocity. These updated parameter values give reasonable fits to the experimental force-velocity data. However, they predict (Table S1) much longer half-time of the displacement associated with the myosin power-stroke than has been observed using optical tweezers (Woody et al., 2019) as well as in response to length steps in muscle cells (Governali et al., 2020). Evaluation of this effect suggests that the rate of the stroke is critically dependent of the total stroke length in the model as well as the fractional sub-division between the first and second sub-stroke (Table S1.

Fig. S1). Fine-tuning was therefore performed to obtain a reasonable fit to both the force-velocity data and the power stroke time course (Model 5 in Table S1). In this process we modified x_2 and reduced the total power-stroke distance from 9 to 8 nm. We also increased the rate constant k_{LH} to 5000 s^{-1} and the free-energy difference ΔG_{PiR} to 5 k_BT from their starting values of 1000 s^{-1} and 1 k_BT, respectively used in all simulations referred to above (Note, our simulations of OM effects below and some other findings later prompted us to change ΔG_{PiR} back to 1 k_BT). We also increased ΔG_{LH} to 11 k_BT from 10 k_BT and changed the ADP dissociation rate constant k_6 back from 75 s^{-1} to 100 s^{-1} (Tables 1-2).

Sensitivity analysis

A key finding was that changes in rather few parameter values modified each of the simulated contractile observables. Particularly, the value of the parameter x_2 (reflecting the subdivision between the first and second sub-stroke if x_3 is constant) critically affects all simulated properties (Fig. S2A-C, E) except the actin-activated ATPase (Fig. S2D). Further, the isometric force and the curvature of the force-velocity relationship (Fig. S2A, C) strongly depend on a similar set of parameters (x_2, x_3, ΔG_{LH}, ΔG_{on}). An increase in ΔG_{on}, leading to an increased attachment rate constant in the model is associated with an increase in both isometric force and a/F_0^* (increased relative power). Changes in the other parameter values on the other hand have qualitatively different effects on isometric force and power. Not unexpectedly, the maximum shortening velocity is primarily dependent on the rate of cross-bridge detachment governed by the slow rate constant k_6. Additionally, the shortening velocity shows dependence on the parameters x_2, and ΔG_{LH} that reflect the magnitude and effectiveness of the power-stroke. Also, self-evidently, the maximum rate of ATP turnover (Fig. S2D) is governed by the slowest process corresponding to myosin attachment to actin determined by the parameter ΔG_{on}. Moreover, a shift of the hydrolysis equilibrium (increase in K_3 by slowing the reverse transition) gives a faster ATPase. The power-stroke half-time, finally, is most sensitive to the value of the parameter x_2 (cf. Fig. S3 for explanation) followed by $\Delta G_{LH.}$

The sensitivity analysis suggests an approach for reducing the modelled isometric force (that was found to be rather high compared to experiments) while increasing the maximum relative power output without affecting other simulated ensemble properties. This could be achieved (Fig. S2A, C) by reducing the power stroke distance (change in x_3 from -8 nm to a less negative value). Whereas such a change would be motivated by the somewhat high isometric force in the model there are uncertainties in the experimental values (see below). Moreover, the required change in x_3 without other changes would make the second sub-stroke (AMD_H -> AMD) appreciably smaller than found in experiments with full-length myosin (Hwang et al., 2021) (although similar to values found in single-headed myosin (Capitanio et al., 2006; Woody et al., 2018a; Woody et al., 2019)). We found in the further analysis of the OM effects below that a substantially reduced free energy difference ΔG_{PiR} (>5-

fold; beyond range of sensitivity analysis) would reduce the isometric force per head while increasing a/F_0*. The latter change is implemented in Table 1 whereas the other options considered in this paragraph
are not. Investigation of the OM effects below are initiated using the same parameter values as used in the sensitivity analysis, i.e. including ΔG_{PiR} = 5 k_BT.

Supplementary Discussion

The discrepancies regarding maximum isometric force between modelling of control conditions and experimental data may be partly attributed to general limitations independent of model as considered in the main paper Discussion. This is highlighted by non-negligible effects of reducing the poorly defined quantity ΔG_{PiR} from 5 to 1 k_BT (Table 3). Moreover, the sensitivity analysis (Fig. S2) showed that small changes in the parameter x_3 would give appreciably lower isometric force with minimal effects on other contractile properties. However, we did not implement the latter change because it would give an appreciably shorter second sub-stroke than suggested by experiments using full-length myosin more like the second sub-stroke from single headed myosin constructs (Woody et al., 2018b).

However, there are also more specific issues that may affect the isometric force. The experimental force per head is calculated from the isometric force per cross-sectional area divided by the total number of myosin heads per cross-sectional area. The latter number, in turn, is calculated from the number of myosin heads per half thick filament (294) (Spudich, 2014) divided by the hexagonal cross-sectional area (1.6 x 10^{-3} μm^2) (Mansson, 2010) surrounding the filament. A discrepancy between model and experiments would result if either the assumptions underlying this calculation or those underlying the modelling are incorrect. One possibility from the model side is that the one-site model is better than the three-site model for cardiac muscle whereas the latter model was favored in our previous modelling of skeletal muscle contraction (Månsson, 2019). A possible reason why the one-site model may be better in cardiac muscle would be if a different activation mechanism (Brunello and Fusi, 2024) only allows binding of myosin heads to the most optimally located sites along a cardiac thin filament. The experimental isometric force may give lower average force per cross-bridge than the model for various reasons. This may include effects of changes in the filament lattice area (due to swelling) in myofibrils as well as the fraction of active heads (e.g. due to heads parked on the thick filament backbone) in the myofilament lattice. Finally, one experimental factor that may give underestimated isometric force at 25 ^{o}C is if the myofibrils and skinned muscle preparations tend to deteriorate at this temperature.

Supplementary Table

Table S1. Optimizing model parameters to fit experimental results

Model version	Model characteristics[a]	Isometric force per head (pN)	Max velocity (nm/s)	a/F_0^*	ATPase	Power-stroke $t_{1/2}$
1a	Tables 1-2, 3d column including k_{LH-}= 1000 s^{-1}; ΔG_{LH}=10 k_BT	1.46 [4.38] [b]	1370	0.047	8.76 s^{-1}	12.6 ms
1b	Tables 1-2, 3d column but k_{LH-}= 5000 s^{-1} and ΔG_{LH}=12 k_BT	1.67 [5.00][b]	1440	0.046	8.77 s^{-1}	0.6 ms
2	As 1a but x_2=-7 nm Total stroke -9 nm	1.04 [3.12][b]	1450	0.061	8.76 s^{-1}	850 ms
3	As 2 but also ΔG_{on} =1.5 k_BT	1.17 [3.51][b]	1430	0.089	14.16 s^{-1}	850 ms
4	As 3 but also k_6=75	1.20 [3.60][b]	1100	0.11	14.15 s^{-1}	850 ms
5 (fine-tuned compromise)	As 4 but x_2=-6 nm. Total stroke -8 nm. ΔG_{LH} =11 k_BT, ΔG_{PiR}=3 k_BT, k_6=100 s^{-1} and k_{LH}= 5000 s^{-1}	1.4 [4.2][b]	1460	0.079	14.16 s^{-1}	0.93 ms
Experiments		1.1 – 2.1[c]	1500-3000[d]	0.06-0.1[e]	10-15 s^{-1} [f]	< 1 ms[g]
6. Other attempt to optimize	As 5 but also ΔG_{on} =2 k_BT	1.54 [4.62] [b]	1470	0.12	22.46 s^{-1}	0.93 ms
7. Other attempt to optimize	As 5 but also ΔG_1 =1 k_BT	1.27 [3.81][b]	1430	0.084	14.15 s^{-1}	1.18 ms

[a]Parameter values suggested by experiments that were changed in modelling. Otherwise both experimental and model parameters as in Tables 1-2.

[b] After multiplication with 3 to account for the possibility that there are 3 sites per 36 nm along actin instead of 1 as assumed in the modelling (Månsson, 2019).

[c]Calculated from data of (Vitale et al., 2021) and (Governali et al., 2020) (slow skeletal muscle) assuming 294 myosin heads per myosin half-filament covering a cross-sectional area of 1.6 x 10^{-15} m^2 and with temperature correction from 15 to 25 °C assuming a Q_{10} of 2.

[d]Data from (Swenson et al., 2017) (Tang et al., 2016; Velayuthan et al., 2023) (Bodt et al., 2024)

[e]Increased a/F_0^* suggests reduced curvature of the force-velocity relationship (with increased relative maximal power). Both parameters a and F_0^* are obtained in fits of Hill´s (Hill, 1938) hyperbolic equation to the data. Experimental data from (Tang et al., 2016; Blair et al., 2020)

[f] Data from (Velayuthan et al., 2023; Berg et al., 2024) (8 s^{-1}) obtained at 23 ºC, assuming Q_{10} = 3.5 (de Tombe and Stienen, 2007) and the presence of some non-functional myosin heads in the solution-based ATPase assay.

[g] Data for low loads from single molecule studies (Woody et al., 2018b; Woody et al., 2019) and also from slow skeletal muscle cells (Governali et al., 2020)

Supplementary Figures

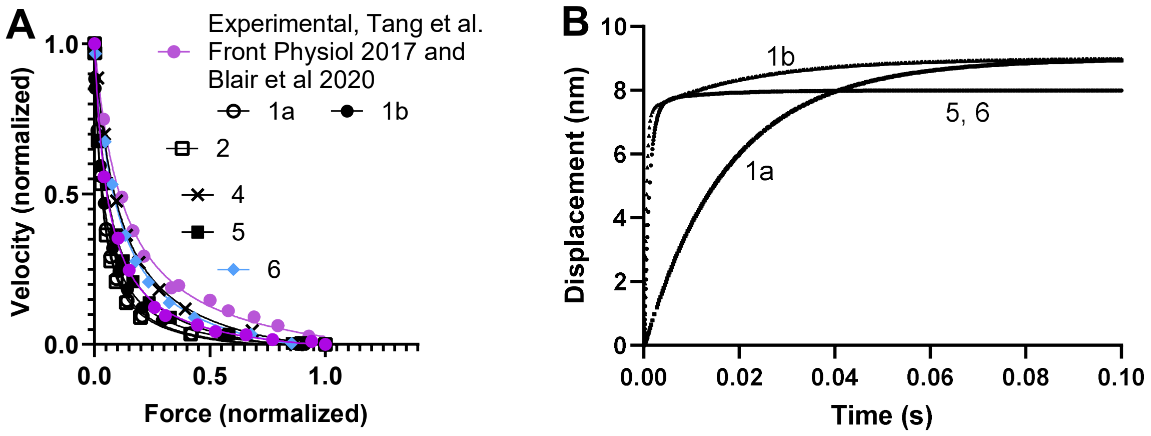

Fig. S1. Effects of changes in key parameter values on the simulated contractile properties. A. Simulated force-velocity relationship corresponding to a large actomyosin ensemble such as a muscle cell under full activation. Simulated data for conditions 1a, 1b, 2, 4, 5 and 6 in Table S1, compared to experimental data (purple) from refs. (Tang et al., 2016) and (Blair et al., 2020). **B.** Displacements simulated under model conditions corresponding to 1a, 1b, 5 and 6 in Table S1. The simulations correspond to averaging of a large number of optical tweezers single molecule mechanics data where attaching motors are allowed to execute largely unhindered power-strokes as in (Woody et al., 2018b). No experimental data given due to somewhat different experimental conditions compared to our simulation conditions. However, the experiments gave half-times of about 1 ms for the early fast recovery, similar to the simulations for conditions 5 and 6.

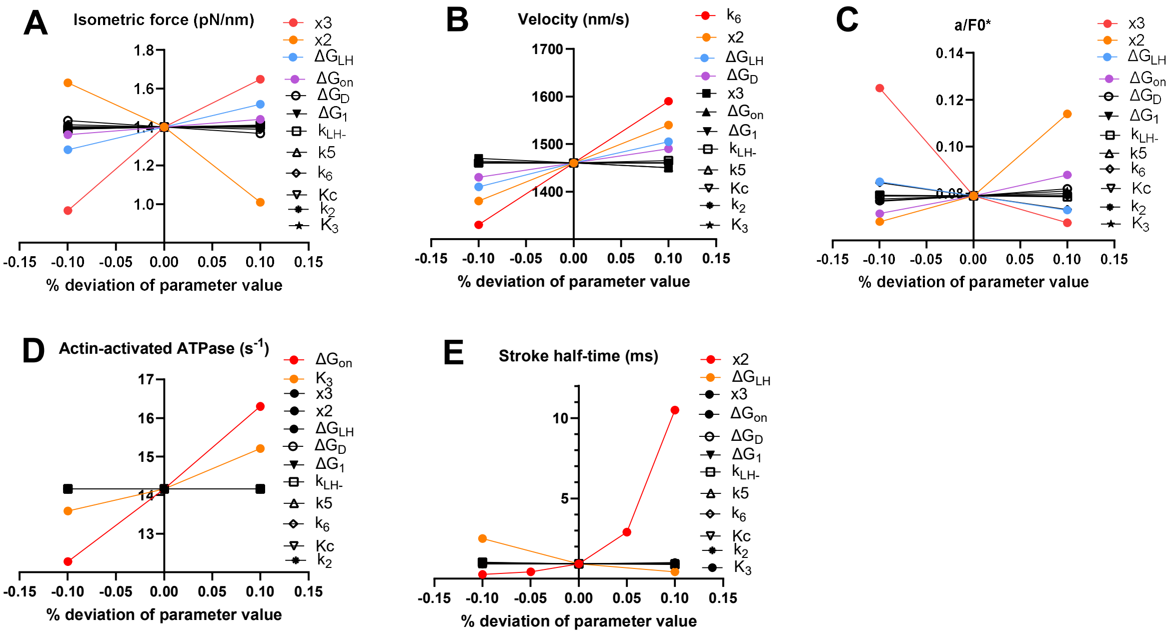

Fig. S2. Simple sensitivity analysis. The graphs show effects of key simulated contractile and kinetic properties on small deviations (± 10 %) of the parameter values from the values used in the present study (Tables 1-2, second column). **A.** Isometric force. The colour coding (from red to orange to blue to purple to black) indicates the most to least critical parameters in determining isometric force in the model. For the parameters x_2 and x_3 a percentage decrease refers to a change to a less negative value, e.g. a 10 % reduction of x_3 refers to a change from -8 to -7.2 nm. **B.** Maximum unloaded shortening velocity. Same principle for color coding as in A. **C.** The ratio a/F_0^* representing the curvature of the force-velocity relationship where increased a/F_0^*reflect reduced curvature and increased maximum relative power output. Same principle for color coding as in A. **D.** Maximum actin-activated ATPase per myosin head (k_{cat}). The colour coding indicates that only two parameters markedly affect k_{cat}. **D.** Half-time ($t_{1/2}$) of displacement (cf. Fig. S1B) corresponding to a myosin power stroke. The colour coding indicates that only two parameters markedly affect $t_{1/2}$. Note, the color coding is used to indicate the relative importance of different parameter. Therefore, a given parameter has different colors in different panels.

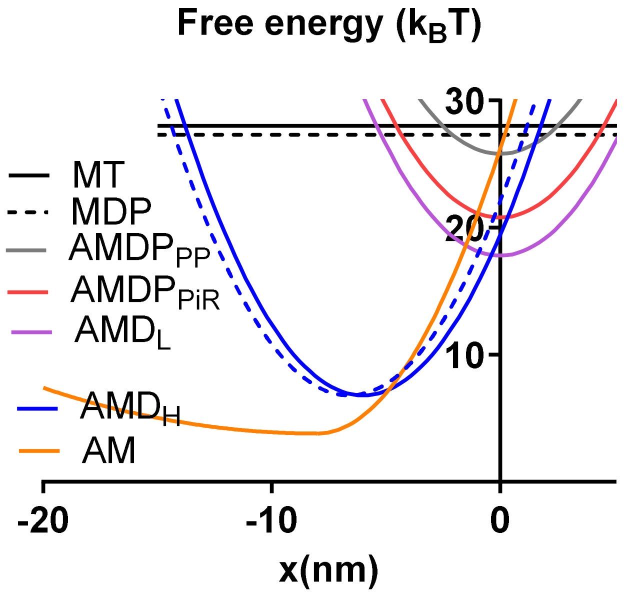

Fig. S3. Shift of the parameter value x_2 by 0.6 nm to more negative value and free energy diagrams like those in Fig. 1B. The small shift of the parameter value x_2 (blue filled to blue dashed line) leads to appreciably increased free energy difference between the AMD_L and the AMD_H state at x=0 nm where the free energy of the AMD_L state is at its minimum. This causes appreciably reduced rate of the power-stroke displacement if this is measured using optical traps with higher stiffness than the motor stiffness of 2.8 pN/nm.

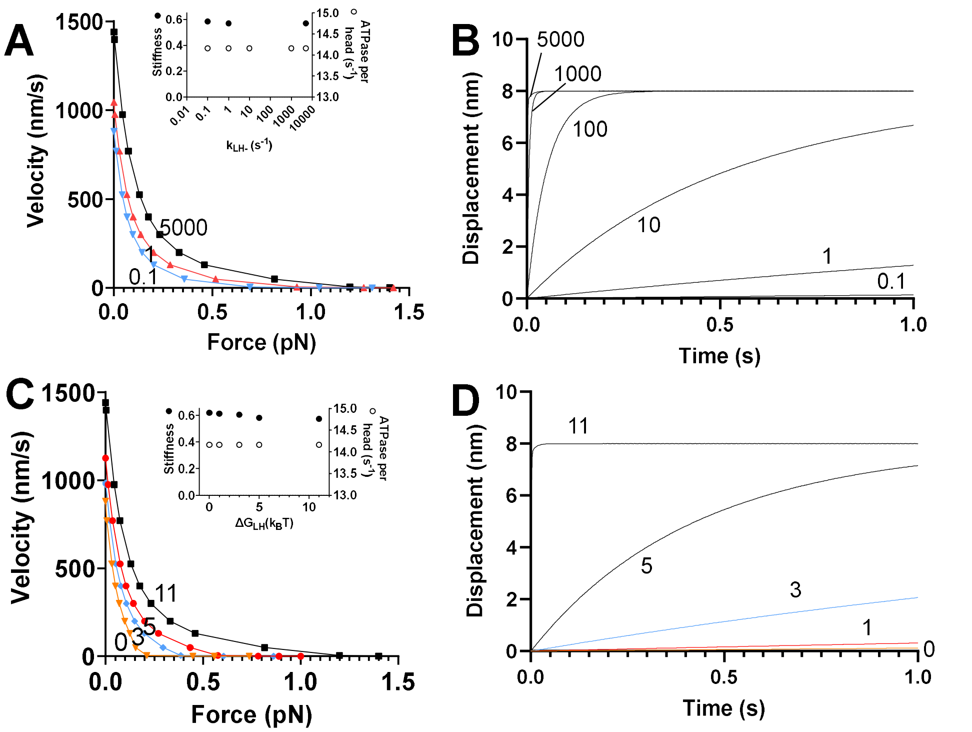

Fig S4. Simulation of isolated effects on force-velocity relationship and power-stroke of reduction in either ΔG_{LH} or k_{LH-}. **A.** Effects on simulated force-velocity relationship of reduction in k_{LH-} from control value of 5000 s^{-1} to either 1 or 0.1 s^{-1} as indicated in the figure. Inset: Number of attached myosin heads in isometric contraction (filled symbols, left vertical axis) and actin activated ATPase (open symbols, right vertical axis) vs k_{LH-}. **B.** Effects on displacement associated with power-stroke of reduction of k_{LH-} from 5000 s^{-1} down to 0.1 s^{-1} and as indicated by numbers in figure. Stiff optical trap assumed (>>3 pN/nm). **C.** Effects on simulated force-velocity relationship of reduction in ΔG_{LH} from control value of 11 k_BT down to 0 k_BT and as indicated by numbers in the figure. Inset: Number of attached myosin heads in isometric contraction (filled symbols, left vertical axis) and actin activated ATPase (open symbols, right vertical axis) vs ΔG_{LH}. **D.** Effects on displacement associated with power-stroke of reduction in ΔG_{LH} from control value of 11 k_BT down to 0 k_BT and as indicated by numbers in figure.

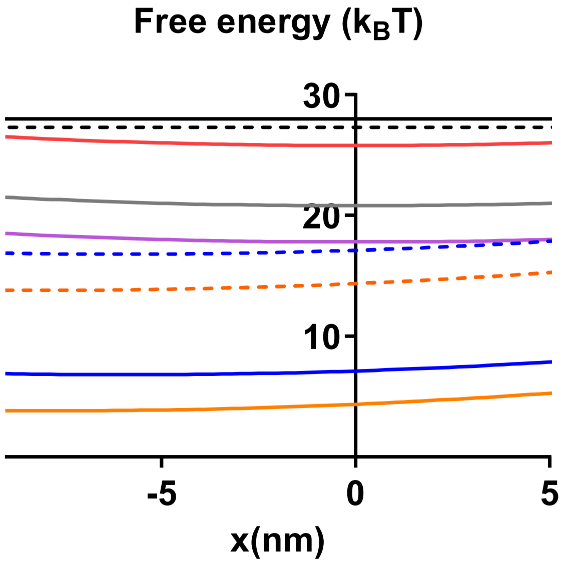

Fig. S5. Free energy diagrams from Fig. 4F, as they are expected to be modified by acting in series with a soft optical trap of stiffness 0.07 pN/nm.

Supplementary References

Berg, A., Velayuthan, L.P., Tagerud, S., Usaj, M., and Månsson, A. (2024). Probing actin-activated ATP turnover kinetics of human cardiac myosin II by single molecule fluorescence. *Cytoskeleton (Hoboken)*. doi: 10.1002/cm.21858.

Blair, C.A., Brundage, E.A., Thompson, K.L., Stromberg, A., Guglin, M., Biesiadecki, B.J., and Campbell, K.S. (2020). Heart Failure in Humans Reduces Contractile Force in Myocardium From Both Ventricles. *JACC Basic Transl Sci* 5(8)**,** 786-798. doi: 10.1016/j.jacbts.2020.05.014.

Bodt, S.M.L., Ge, J., Ma, W., Rasicci, D.V., Desetty, R., McCammon, J.A., and Yengo, C.M. (2024). Dilated cardiomyopathy mutation in beta-cardiac myosin enhances actin activation of the power stroke and phosphate release. *PNAS Nexus* 3(8)**,** pgae279. doi: 10.1093/pnasnexus/pgae279.

Brunello, E., and Fusi, L. (2024). Regulating Striated Muscle Contraction: Through Thick and Thin. *Annu Rev Physiol* 86**,** 255-275. doi: 10.1146/annurev-physiol-042222-022728.

Capitanio, M., Canepari, M., Cacciafesta, P., Lombardi, V., Cicchi, R., Maffei, M., et al. (2006). Two independent mechanical events in the interaction cycle of skeletal muscle myosin with actin. *Proceedings of the National Academy of Sciences of the United States of America* 103(1)**,** 87-92.

de Tombe, P.P., and Stienen, G.J. (2007). Impact of temperature on cross-bridge cycling kinetics in rat myocardium. *J Physiol* 584(Pt 2)**,** 591-600. doi: 10.1113/jphysiol.2007.138693.

Governali, S., Caremani, M., Gallart, C., Pertici, I., Stienen, G., Piazzesi, G., et al. (2020). Orthophosphate increases the efficiency of slow muscle-myosin isoform in the presence of omecamtiv mecarbil. *Nat Commun* 11(1)**,** 3405. doi: 10.1038/s41467-020-17143-2.

Hill, A.V. (1938). The heat of shortening and the dynamic constantsof muscle. *Proceedings of the Royal Society B 136–195* 126**,** 136-195.

Hwang, Y., Washio, T., Hisada, T., Higuchi, H., and Kaya, M. (2021). A reverse stroke characterizes the force generation of cardiac myofilaments, leading to an understanding of heart function. *Proc Natl Acad Sci U S A* 118(23). doi: 10.1073/pnas.2011659118.

Månsson, A. (2010). Actomyosin-ADP states, inter-head cooperativity and the force-velocity relation of skeletal muscle. *Biophysical Journal* 98**,** 1237-1246.

Månsson, A. (2019). Comparing models with one versus multiple myosin-binding sites per actin target zone: The power of simplicity. *J Gen Physiol* 151**,** 578-592. doi: 10.1085/jgp.201812301.

Spudich, J.A. (2014). Hypertrophic and dilated cardiomyopathy: four decades of basic research on muscle lead to potential therapeutic approaches to these devastating genetic diseases. *Biophys J* 106(6)**,** 1236-1249. doi: 10.1016/j.bpj.2014.02.011.

Swenson, A.M., Tang, W., Blair, C.A., Fetrow, C.M., Unrath, W.C., Previs, M.J., et al. (2017). Omecamtiv Mecarbil Enhances the Duty Ratio of Human beta-Cardiac Myosin Resulting in Increased Calcium Sensitivity and Slowed Force Development in Cardiac Muscle. *J Biol Chem* 292(9)**,** 3768-3778. doi: 10.1074/jbc.M116.748780.

Tang, W., Blair, C.A., Walton, S.D., Malnasi-Csizmadia, A., Campbell, K.S., and Yengo, C.M. (2016). Modulating Beta-Cardiac Myosin Function at the Molecular and Tissue Levels. *Front Physiol* 7**,** 659. doi: 10.3389/fphys.2016.00659.

Velayuthan, L.P., Moretto, L., Tagerud, S., Usaj, M., and Månsson, A. (2023). Virus-free transfection, transient expression, and purification of human

cardiac myosin in mammalian muscle cells for biochemical and biophysical assays. *Sci Rep* 13(1)**,** 4101. doi: 10.1038/s41598-023-30576-1.

Vitale, G., Ferrantini, C., Piroddi, N., Scellini, B., Pioner, J.M., Colombini, B., et al. (2021). The relation between sarcomere energetics and the rate of isometric tension relaxation in healthy and diseased cardiac muscle. *J Muscle Res Cell Motil* 42(1)**,** 47-57. doi: 10.1007/s10974-019-09566-2.

Woody, M.S., Capitanio, M., Ostap, E.M., and Goldman, Y.E. (2018a). Electro-optic deflectors deliver advantages over acousto-optical deflectors in a high resolution, ultra-fast force-clamp optical trap. *Opt Express* 26(9)**,** 11181-11193. doi: 10.1364/OE.26.011181.

Woody, M.S., Greenberg, M.J., Barua, B., Winkelmann, D.A., Goldman, Y.E., and Ostap, E.M. (2018b). Positive cardiac inotrope omecamtiv mecarbil activates muscle despite suppressing the myosin working stroke. *Nat Commun* 9(1)**,** 3838. doi: 10.1038/s41467-018-06193-2.

Woody, M.S., Winkelmann, D.A., Capitanio, M., Ostap, E.M., and Goldman, Y.E. (2019). Single molecule mechanics resolves the earliest events in force generation by cardiac myosin. *Elife* 8. doi: 10.7554/eLife.49266.
